# Bioimpedance: From Body Composition Assessment to Emerging Technologies in Clinical Nutrition

**DOI:** 10.3390/nu18172889

**Published:** 2026-09-03

**Authors:** Ruth Nekane Pita, Isabel María Fernández, Oibar Martínez, Jose Miguel Miranda

**Affiliations:** 1 Department EMFTEL, Faculty of Physics, University Complutense of Madrid, 28040 Madrid, Spain; isabef07@ucm.es (I.M.F.); miranda@ucm.es (J.M.M.); 2 Higher School of Experimental Sciences and Technology, Rey Juan Carlos University, 28933 Mostoles, Spain; oibar.vilchez@urjc.es

**Keywords:** bioelectric impedance, BIA, PhA, BIVA, body composition, clinical nutrition, sarcopenia, obesity, hydration status, bioimpedance wearables

## Abstract

This review examines the physical foundations, predictive models, emerging technologies and recent research trends in bioimpedance for clinical nutrition. A bibliometric analysis of cumulative scientific production from 1980 to 2025 shows that weighted logistic models provide a good descriptive fit to the evolution of the literature (R^2^ ≥ 0.999). Bioimpedance research remains in an expanding phase, with its projected inflection point lying beyond 2026. These fits are descriptive and model-dependent: high coefficients of determination on cumulative counts are not in themselves evidence of predictive validity, and inflection points beyond the observation window are extrapolations. We introduce the Measurement-Independent Factor (MIF), which isolates the anthropometric contribution from the bioimpedance measurement in predictive equations. Phase Angle (PhA) has emerged as the most extensively studied bioimpedance descriptor, since it avoids the population-specific prediction equations required for estimates such as FFM or FM, although its absolute value remains dependent on measurement conditions, instrumentation and biological characteristics. Body-composition estimates obtained with devices from different manufacturers should not be interpreted interchangeably. Electrode technologies, bioimpedance wearables, and bioimpedance imaging are reviewed as major emerging research directions. A second bibliometric analysis covering the most-cited literature from 2020 to 2026 and 1655 original articles published during 2025–2026 show that recent research focuses primarily on obesity and adiposity, together with sarcopenia and frailty, whereas critical and acute care remain underrepresented.

## 1. Bioimpedance in Nutrition

### 1.1. Scope, Terminology, and Organization

Bioelectrical impedance analysis (BIA) is currently one of the most widely used techniques for assessing body composition and indirectly estimating basal metabolic rate (BMR). Its widespread adoption is attributable to a unique combination of four major characteristics rarely found together in physiological measurements: non-invasive nature, portability, ease of operation, and low cost. BIA provides the estimates of several physiologically relevant body compartments, including total body water (TBW), fat-free mass (FFM), fat mass (FM), extracellular water (ECW), and intracellular water (ICW). Beyond conventional BIA, two major branches of bioimpedance emerged: bioimpedance spectroscopy (BIS), for characterizing tissue dielectric properties and body fluid distribution, and electrical impedance tomography (EIT), for functional conductivity imaging.

This work is a narrative methodological review. All clinical evidence discussed in the following sections is drawn from the published literature. Three analyses were generated specifically for this manuscript: the bibliometric assessment of publication activity described in Section 1.3, the algebraic decomposition of published prediction equations introduced as the measurement-independent factor (MIF), and the Cole model simulation of Section 2.4, fitted to public bioimpedance records. All three are exploratory and descriptive in nature, and are presented as illustrative analyses rather than as validation studies.

Three terms are used throughout this review. A reference method is a technique used to calibrate or benchmark another. A criterion method is the technique taken as comparator in a given validation study. A validated method is one whose agreement with such a comparator has been quantified in the population of interest. BIA is treated here as a validated method for specific populations and applications, and not as a reference method.

The review is addressed to readers from both clinical nutrition and biomedical engineering, and is organized as follows. The historical development of the technique is outlined first, followed by the growth of the corresponding literature. Section 2 then presents the physical basis of the measurement. Two points receive particular attention there: the complex phasor representation of impedance and the capacitive behavior of cell membranes. Neither is always stated explicitly in the recent clinical literature, although both help to interpret what is measured. The conversion of electrical quantities into body composition estimates and the limits of that conversion are examined in Section 3. Emerging technologies related to those limits follow in Section 4. The recent literature is considered the last. Among the most-cited work published between 2020 and 2026, sustained attention is apparent in a limited number of clinical areas. Citation counts, however, need time to accumulate. Non-review articles from 2025 to 2026 are therefore examined separately at the end of Section 5. That comparison offers a tentative indication of which of the gaps noted above are being addressed.

### 1.2. First Steps

The origins of bioimpedance date back to the nineteenth century, when investigators first recognized that biological tissues exhibit frequency-dependent electrical properties [1]. The theoretical description of dielectric dispersion that followed provided the physical framework underpinning impedance spectroscopy [2]. Early studies demonstrated that tissues containing high concentrations of water and electrolytes, such as blood and skeletal muscle, conduct electrical current far more readily than adipose tissue or bone. Thomasset reported electrical impedance measurements with needle electrodes to determine TBW [3] and was the first to employ two measurement frequencies (1 and 100 kHz) to estimate ECW [4]. Nyboer then introduced the tetrapolar surface-electrode configuration for bioimpedance measurements [5], while Hoffer et al. demonstrated a strong correlation between whole-body bioimpedance and TBW, validating the method against tritium dilution techniques [6].

During the 1980s, Lukaski et al. showed that whole-body electrical impedance could be used to accurately estimate FFM [7], subsequently validating the tetrapolar impedance method against these reference techniques [8]. Concurrently, Kushner and Schoeller established that the underlying physical basis of this estimation relies on the strong correlation between TBW and the impedance index, mathematically expressed as height squared divided by resistance [9]. Further studies combined raw electrical measurements with anthropometric variables in multivariate regression models, improving predictive accuracy across diverse populations [10]. However, Deurenberg et al. documented the errors that arise when equations developed in one population are applied to subjects differing in age, sex or body composition [11]. The National Institutes of Health Technology Assessment Conference reinforced these observations, emphasizing that the validity of BIA predictions depends on the characteristics of the calibration cohort and on key physiological assumptions of the method. The chief among these is the assumption that the hydration fraction of FFM remains constant at approximately 73.2% in healthy, normohydrated subjects [12]. Sun et al. later improved the basic BIA 2-component model (fat and fat-free) with a multicomponent reference method accounting for variations in mineral bone, water, and fat [13].

To overcome limitations in fluid distribution assessment and regional body composition, Ward et al. further developed multifrequency BIA (MF-BIA), in which impedance is measured at several discrete frequencies to improve the characterization of body fluid compartments [14]. This development was soon complemented by segmental BIA, which extended impedance measurements to obtain direct information from limbs and trunk [15].

Bioimpedance research expanded beyond whole-body composition analysis toward imaging techniques capable of reconstructing the spatial distribution of tissue electrical properties. EIT was originally inspired by geophysical electrical prospecting methods. It was introduced for medical applications by Barber, Brown, and co-workers, who developed one of the first systems capable of generating cross-sectional conductivity images from surface-electrode measurements [16]. Their initial work identified the thorax as the most promising clinical target because of the large conductivity contrast between air-filled lungs, blood, and surrounding tissues.

Cheney, Isaacson, and Newell presented a comprehensive description of the physical and mathematical foundations of EIT together with the Adaptive Current Tomography system [17]. These theoretical developments were further consolidated by Borcea, who reviewed the inverse conductivity problem [18]. EIT evolution from an experimental imaging modality to a clinically useful bedside monitoring technique was reviewed by Adler and Holder [19], and Bodenstein et al., who focused on mechanically ventilated patients [20].

BIS emerged progressively from early bioimpedance developments [21]. Methodological analyses have shown that BIS provides greater biological insight than conventional regression-based BIA [22]. This advantage arises because BIS relies on electrical models of tissue behavior rather than solely on statistical prediction equations. By extrapolating the measured spectrum to extreme frequencies (*f* → 0, *f* → ∞), BIS estimates ECW and TBW from electrically meaningful parameters. This approach reduces dependence on arbitrary measurement frequencies and yields a more physiologically grounded description of body fluid distribution.

Bioelectrical Impedance Vector Analysis (BIVA) technique is attributed to Piccoli et al., who first described the method in 1994 [23]. In a second study, the same group defined the bivariate reference tolerance ellipses that transformed BIVA into a practical tool for the assessment of hydration status and body cell mass (BCM) in both healthy subjects and patients [24]. While traditional BIA relies on empirical regression equations, BIVA directly analyzes the raw impedance vector plotted on a resistance–reactance graph. BIVA has been proven highly valuable in clinical conditions associated with altered hydration and body composition, including severe obesity, where conventional regression equations often provide inaccurate estimates [25].

The 2004 guidelines of the European Society for Clinical Nutrition and Metabolism (ESPEN) played a major role in standardizing and consolidating the clinical application of BIA. Before these consensus documents, the clinical implementation of BIA remained heterogeneous, with considerable variability in measurement protocols and interpretation across institutions. The first ESPEN guideline synthesized the theoretical foundations, methodological requirements, and technical limitations of BIA, providing a standardized scientific framework for its clinical application [26]. The companion paper translated these principles into evidence-based clinical recommendations [27].

Reference methods evolved from hydrodensitometry and direct anthropometry [28], toward more accurate techniques, including isotope dilution, neutron activation analysis, magnetic resonance imaging and computed tomography [29]. Dual-energy X-ray absorptiometry (DXA) has become one of the most popular reference methods during the last 25 years [30].

Three distinct uses of BIA are separated throughout this review. The first is the estimation of absolute compartment values, which depends entirely on prediction equations and on the population in which they were calibrated. The second is the intraindividual monitoring of change over time, in which a stable equation bias largely cancels, and the relevant quantity is the difference rather than the absolute value. The third is the direct interpretation of raw bioelectrical parameters, such as PhA and BIVA, which requires no regression step. Most of the discrepancies discussed in the following sections concern the first use only, and a conclusion established for one of the three does not transfer to the others.

### 1.3. Bibliometric Search Strategy

The evolution of research activity in body composition assessment within the field of nutrition was studied through a systematic literature search. This search was performed on 22 July 2026 using the Web of Science (WoS) Core Collection database, with all ten editions available under our institutional subscription selected: Science Citation Index Expanded, Social Sciences Citation Index, Arts & Humanities Citation Index, Conference Proceedings Citation Index—Science, Conference Proceedings Citation Index—Social Sciences & Humanities, Book Citation Index—Science, Book Citation Index—Social Sciences & Humanities, Emerging Sources Citation Index, Current Chemical Reactions, and Index Chemicus. No restriction on publication year was applied at the search stage, and the analysis reported here covers the period 1980–2025. The analysis included five of the most widely used instrumental techniques for body composition assessment: BIA, DXA, computed tomography (CT), magnetic resonance imaging (MRI), and ultrasound (US). A second, separate corpus is analyzed in Section 5: it applies the same search equations to the 2020–2026 window, without the restriction to the Article document type, and retains only publications above a citation-rate threshold. Its counts are therefore neither a subset of nor comparable with those reported here.

Scientific terminology is not fully standardized; therefore, a simple keyword search may miss a substantial number of relevant publications. To overcome this limitation, dedicated search equations were designed for each modality. Each equation combines the principal terms and accepted synonyms of the corresponding technique. These were then combined with a common set of nutrition-related terms using the Topic Search (TS) field of the WoS database. Table 1 shows the complete search equations. The inclusion of alternative terminology, including acronyms, full technique names and common descriptors, was intended to improve sensitivity without sacrificing specificity. This strategy reduces the risk of excluding relevant studies due to nomenclature differences across journals and research communities.

Three main criteria governed the design of the equations. First, the five technique blocks were built to a comparable level of specificity. Generic expressions such as “magnetic resonance” also retrieve spectroscopy and non-imaging applications, so they were replaced by explicit imaging terms. Second, phrases were truncated inside quotation marks rather than left unquoted. WoS applies automatic lemmatization to unquoted Topic terms but disables it within exact phrases, so internal truncation preserves morphological variants (impedance/impedances, absorptiometry/absorptiometric, tomography/tomographic) while retaining the precision of the phrase. Third, acronyms were admitted only where unambiguous in this context. DXA, DEXA, MRI, BIA and BIVA were retained because the mandatory conjunction with the nutrition block confines them to their intended meaning. The isolated acronym CT was excluded, since it also denotes the cycle threshold of quantitative PCR and other unrelated concepts; bounded forms were used instead.

Two domain-specific safeguards were applied. In the BIA equation, “phase angle” is retrieved only in conjunction with a bioimpedance term, which excludes the extensive electrical-engineering and gait-analysis literature that uses the same expression with an unrelated meaning. In the US equation, an exclusion clause removes ultrasound-assisted extraction and sonication, which is a large body of food-technology work that satisfies the nutrition block without measuring body composition; this contaminant is specific to US, and retaining it would have inflated that series alone. The nutrition block deliberately omits “body composition” as a search term, because it is a methodological descriptor rather than a marker of the clinical nutrition domain. Its inclusion would disproportionately expand the CT and MRI series through the radiological and oncological literature, again at the expense of comparability.

All five queries were executed with the aim of reflecting publication activity rather than retrieval settings. Searches were therefore restricted to the Article document type, which excludes reviews. Reviews are commissioned and published at rates set by editorial dynamics that differ between modalities. Including them would therefore inflate the more established techniques. The Article document type is also the one most consistently assigned across the full time window.

**Figure 1:**
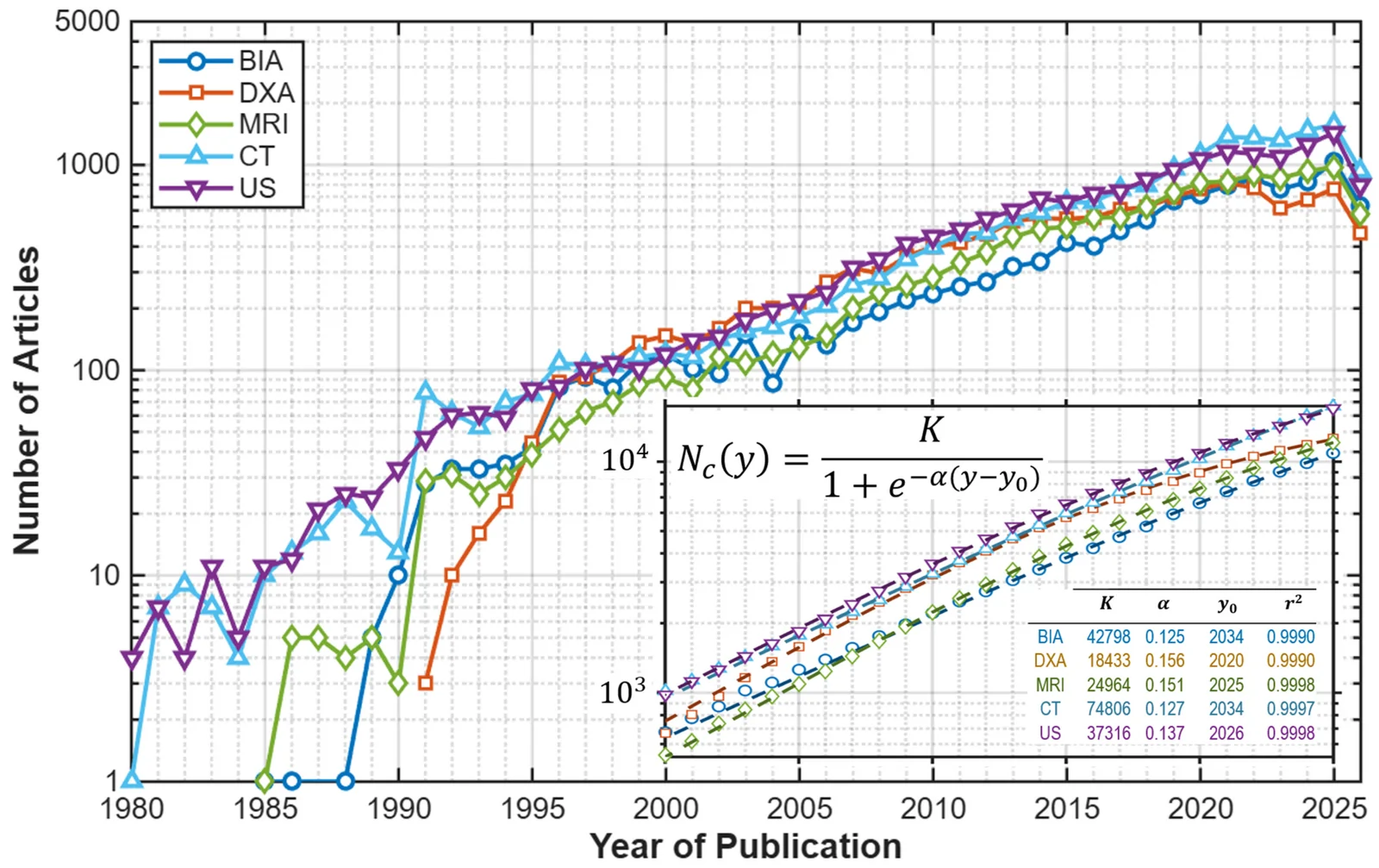
Annual scientific production related to the five principal body composition assessment techniques between 1980 and June 2026. Main panel: annual number of publications. Inset: cumulative publication counts over 2000–2025 ($N_{c}$), with logistic models fitted to the complete 1980–2025 record. *K*: asymptotic publication level predicted by the model (cumulative number of publications); *α*: intrinsic growth rate (year^−1^); *y*_0_: inflection year corresponding to the maximum growth rate (calendar year); *r*^2^: coefficient of determination (dimensionless). Model parameters were estimated by weighted non-linear least squares, applying exponentially increasing weights to emphasize the most recent years while preserving the complete publication history.

The retrieval procedure therefore involved no screening stage beyond the search itself. For each of the five techniques, the corresponding equation was run in the Topic Search field, restricted to the Article document type, and the complete record set was exported directly from the Web of Science interface without further selection. The annual and cumulative counts derived from these exports are those plotted in Figure 1. No record was excluded manually or automatically at any subsequent stage.

### 1.4. Evolution of Research Activity, 1980–2025

Figure 1 shows the results of the bibliometric search from the WoS Core Collection. The bibliometric data show that research volume in body composition techniques has increased steadily since 1980. Their trajectories, however, reflect different lifecycles. CT and MRI grew exponentially during the 1990s and established themselves as laboratory reference methods. In contrast, BIA has evolved from a basic predictive tool into a widely used methodology for nutritional assessment.

BIA publications experienced a sharp acceleration during the 1990s. This growth was driven by the transition from single-frequency empirical equations to multifrequency models, alongside the validation of raw electrical parameters, such as PhA. DXA entered the literature later (1991) and quickly reached a plateau of 700–900 annual articles as the standard for the three-compartment model. BIA, in contrast, maintained a continuous upward trajectory and reached a peak of 1039 articles in 2025. Notably, the publication volume and growth rate of BIA are fully comparable to, and in some years exceed, those of DXA, CT and MRI.

The inset of Figure 1 presents the cumulative scientific production alongside weighted logistic growth models fitted to the 1980–2025 period but was displayed for 2000–2025 for visual clarity. Model parameters were estimated independently for each technique by minimizing a weighted least-squares objective function, with cumulative counts normalized prior to optimization. To characterize the current maturity of each research field rather than its historical emergence, residuals were weighted using an exponential function that progressively increased the contribution of more recent years. This weighting reduced the influence of the sparse publication counts observed during the early stages of each field while preserving the information contained in the complete historical record. The weight assigned to year t was w(t) = exp[λ(t − 1980)], with a weighting rate λ = 0.03 yr^−1^. This rate is distinct from the intrinsic growth rate α of the logistic model. Weights were normalized to unity at the first year of the series. The relative contribution of a residual therefore increased monotonically by a factor of approximately 3.9 between 1980 and 2025. This value of λ is not a standard constant, and it was fixed a priori rather than estimated from the data. It corresponds to a weight doubling time of ln2/λ ≈ 23 years, about half of the 46-year window, so that the mature phase of each field carries roughly four times the weight of its emergence while no year is discarded.

Sensitivity to the weighting and to the functional form was assessed by refitting all five series. Varying the weighting rate λ between zero and 0.06 yr^−1^ displaced the inflection year of BIA by 1.6 years in total, from 2032.5 to 2034.1, and changed its asymptote by less than 8%. Variations were smaller for the remaining techniques. The choice of λ therefore does not drive the conclusions. Replacing the logistic by a Gompertz model changed the estimated asymptotes far more substantially. For BIA and CT, the two series whose inflection lies beyond the observation window, the asymptote became effectively non-identifiable. K reached 1.9 × 10^7^ and 1.3 × 10^8^. The estimated inflection years moved into the twenty-second century. The confidence intervals exceeded the estimates themselves, and the Jacobian was numerically singular. The ordering of the five techniques by inflection year was nevertheless identical under both functional forms, from DXA and MRI through US to BIA and CT. The interpretation offered here therefore rests on that ordering and on the relative maturity it implies, and not on the absolute values of K or α. For the pre-inflection series, those values are governed by the functional form assumed rather than by the observed record.

The logistic model provided an excellent description of the cumulative publication trends for all five techniques, with coefficients of determination equal to or above 0.999. Because cumulative counts are monotonic and highly structured variables, such values indicate a good descriptive fit rather than predictive validity. The estimated intrinsic growth rates α indicate that DXA expanded most rapidly over the analyzed period, whereas MRI and ultrasound showed intermediate rates and BIA and CT showed more gradual but sustained growth. MRI and US yielded inflection points close to the present, at y_0_ = 2025.4 ± 0.5 and 2026.4 ± 0.7, respectively. In contrast, BIA and CT produced substantially later inflection years, 2033.6 ± 2.8 and 2034.4 ± 1.7, whereas y_0_ for DXA was the earliest, at 2020.2 ± 0.7. Because the inflection years estimated for BIA and CT lie beyond the observation window, the corresponding K values should be interpreted as extrapolations rather than as observed saturation levels. All intervals reported here are nominal 95% weighted least-squares confidence intervals obtained from the Jacobian at the optimum.

## 2. Physical Principles

### 2.1. Why Is Bioimpedance a Complex Number?

Bioimpedance (***Z***) is a complex quantity defined as the ratio of the voltage phasor (***V***) to the electric current phasor (***I***):

(1) Z=V/I

Phasors are complex-number representations of time-harmonic sinusoidal variables. They allow us to retrieve the time-dependent function they represent by multiplying the phasor by the complex exponential factor *exp* (*jωt*) and extracting the real part:

(2) v(t)=ReV⋅ejωt=ReVmejθv⋅ejωt=Vmcos(ωt+θv)

(3) i(t)=ReI⋅ejωt=ReImejθi⋅ejωt=Imcos(ωt+θi)

where $V_{m}$ and $I_{m}$ are the peak amplitudes of the voltage and current, respectively, ω is the angular frequency (ω=2πf), $\theta_{v}$ and $\theta_{i}$ are the phases of voltage and current, respectively, and j is the imaginary unit ($j=\sqrt{-1}$). Complex impedance can be separated into its real and imaginary parts as

(4) Z=R+jX

The physical interpretation of these components determines how energy interacts with the medium:

○ **Resistance.** Quantifies the dissipation of electrical energy as heat. In biological tissues this dissipation is mostly due to collisions of ions moving through intra- and extracellular fluids. It is always a positive quantity.
○ **Reactance.** Quantifies the storage and release of electromagnetic energy within the system and can be either positive or negative. The sign depends entirely on the stored energy: a positive reactance indicates energy storage in a magnetic field, whereas a negative reactance indicates storage in an electric field. In circuital terms, it is customary to represent a positive reactance as an inductor and a negative one as a capacitor. From the circuit theory, it follows that the reactance of a pure inductor with inductance L is $X_{L}=L\omega$, whereas the reactance of a pure capacitor with capacitance C is $X_{C}=-1/\left(\omega C\right)$.

In some applications, bioimpedance can also be expressed in terms of the modulus |Z| and phase angle $\varphi_{A}$,

(5) $$Z=|Z|e^{j\varphi_{A}}$$

where $\left|Z\right|$ = $\sqrt{R^{2}+X^{2}}$; and $\varphi_{A}=\theta_{v}-\theta_{i}$.

Biological tissues store energy almost exclusively within an electric field. Because this capacitive storage of electrical energy is heavily dominant over any negligible magnetic inductive properties, bioimpedance characteristically exhibits a negative reactance. However, the imaginary part is typically small over the frequency range employed in BIA. For this reason, a number of analytical models treat each segment as a purely resistive element and neglect the phase contribution. Accordingly, the argument of ***Z*** in Equation (4) is negative for a capacitive biological load. The clinical phase angle reported throughout this review follows the positive BIA convention, PhA = arctan(|X|/R), and is thus the negative of that argument.

### 2.2. Segmental Bioimpedance

**Figure 2:**
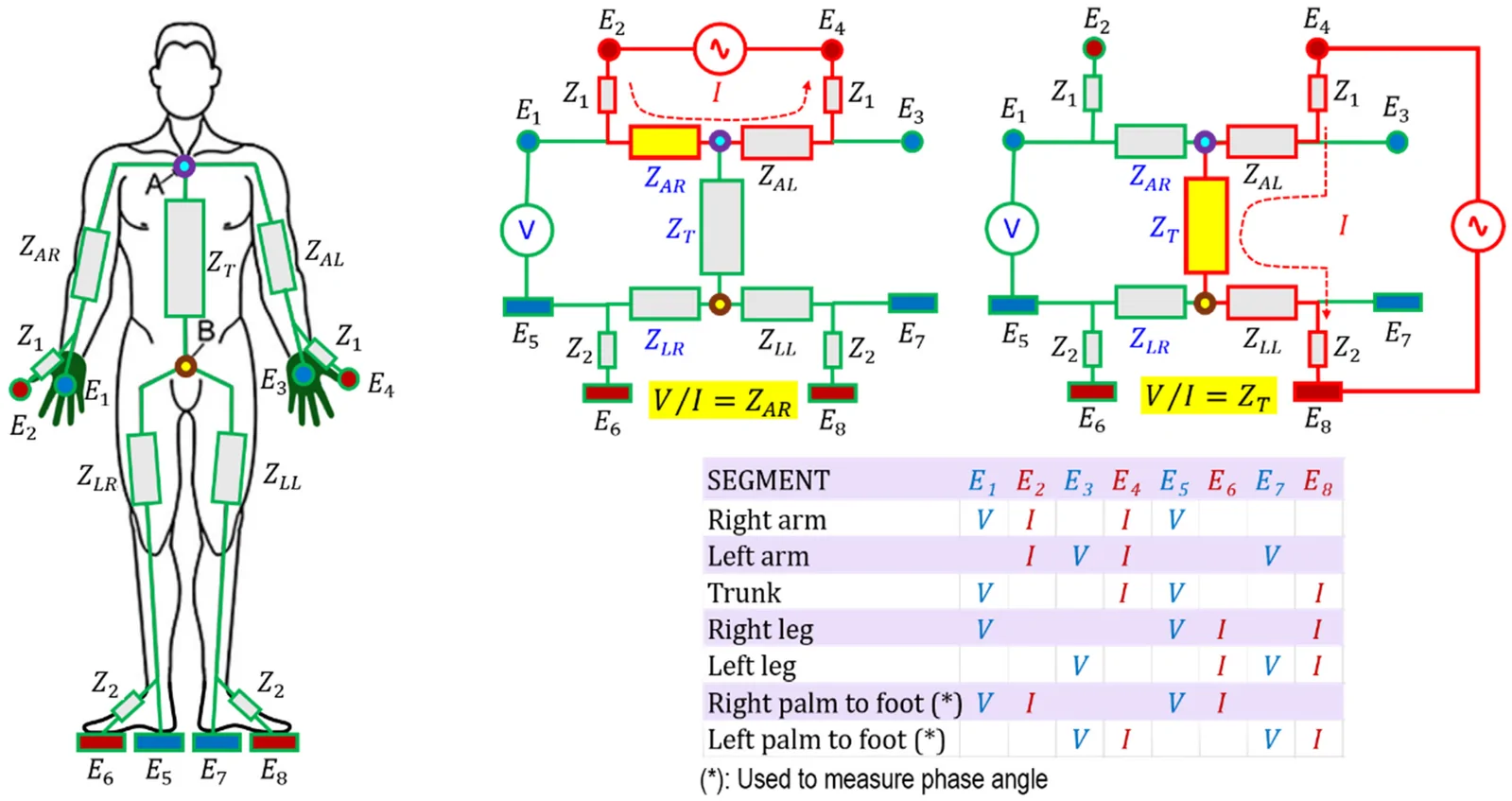
Electrode placement for segmental bioimpedance measurements (**left**), together with the corresponding equivalent electrical circuits for the right arm (**center**) and trunk (**right**). When no current flows through a circuit element, its voltage drop is zero. The auxiliary table summarizes the combinations of current-injecting and voltage-sensing electrodes used to measure each body segment. Adapted from [31]. All impedance, voltage, and current quantities are represented as complex numbers.

Segmental bioimpedance analysis (SBIA) is based on the measurement of individual body segment impedances. Compared to conventional whole-body bioimpedance analysis, SBIA provides more localized information about tissue composition and physiological changes, allowing independent assessment of each limb and trunk. Figure 2 illustrates the electrode arrangement and the equivalent electrical circuits for representative measurements.

The measurements are performed using the tetrapolar method, which minimizes the influence of the electrode–skin interface impedance. Although the measurement instrument incorporates eight surface electrodes (*E*_1_–*E*_8_), only four are active during each acquisition. An electronic switching network automatically selects the appropriate electrodes depending on the body segment to be measured.

As summarized in the auxiliary table, two electrodes are used to inject a sinusoidal current (*I*), while two different electrodes are employed to measure the resulting voltage drop (*V*). Since the voltage-sensing circuit has a very high input impedance, virtually no current flows through the voltage electrodes, making the measured voltage independent of the contact impedance at these electrodes. Consequently, the calculated impedance is determined almost exclusively by the electrical properties of the selected body segment.

The electrode selection depends on the anatomical region under investigation. For example, when the left arm is measured, current is injected through electrodes *E*_2_ and *E*_4_, whereas the voltage is sensed between *E*_3_ and *E*_7_. Similarly, different electrode combinations are automatically selected for the trunk, each leg, or palm-to-foot measurements for full body assessment. This switching strategy enables all segmental measurements to be performed without repositioning the electrodes, ensuring repeatability and fast acquisition.

The usefulness of the tetrapolar method becomes evident when analyzing the equivalent circuits. As an example, the equivalent circuit shown on the left of Figure 2 represents the measurement of the right arm. The excitation current is injected through the upper electrodes and flows through the arm segment under investigation. Because the voltmeter draws negligible current, no significant voltage drop is produced across the voltage electrode contact impedances. Furthermore, the current flowing through the trunk and the remaining limbs produces only a negligible contribution to the measured voltage because the voltage electrodes are positioned immediately across the right arm. Under these conditions, the measured impedance is *Z*_AR_. A similar principle applies to trunk measurements, illustrated in the right-hand equivalent circuit of Figure 2.

*Z*_1_ and *Z*_2_ represent the local tissue impedances between the current-injecting electrode and the voltage-sensing electrode located on the same hand or foot. Since these impedances lie exclusively within the current injection path, they are connected in series with the body segment under investigation and carry the same excitation current. However, they are located outside the voltage measurement terminals and therefore do not contribute to the measured voltage. Consequently, neither *Z*_1_ nor *Z*_2_ affects the measured impedance. These impedances must remain finite rather than being assumed equal to zero. Otherwise, the current-injecting and voltage-sensing electrodes would become electrically equivalent, effectively reducing the measurement to a two-terminal (bipolar) configuration. If a conventional two-electrode configuration was used, where the same pair of electrodes injects the current and measures the resulting voltage drop, the high impedance of the skin–electrode interface would dominate the measurement, masking the impedance contributions of the internal tissues.

### 2.3. Body Composition Compartments

**Figure 3:**
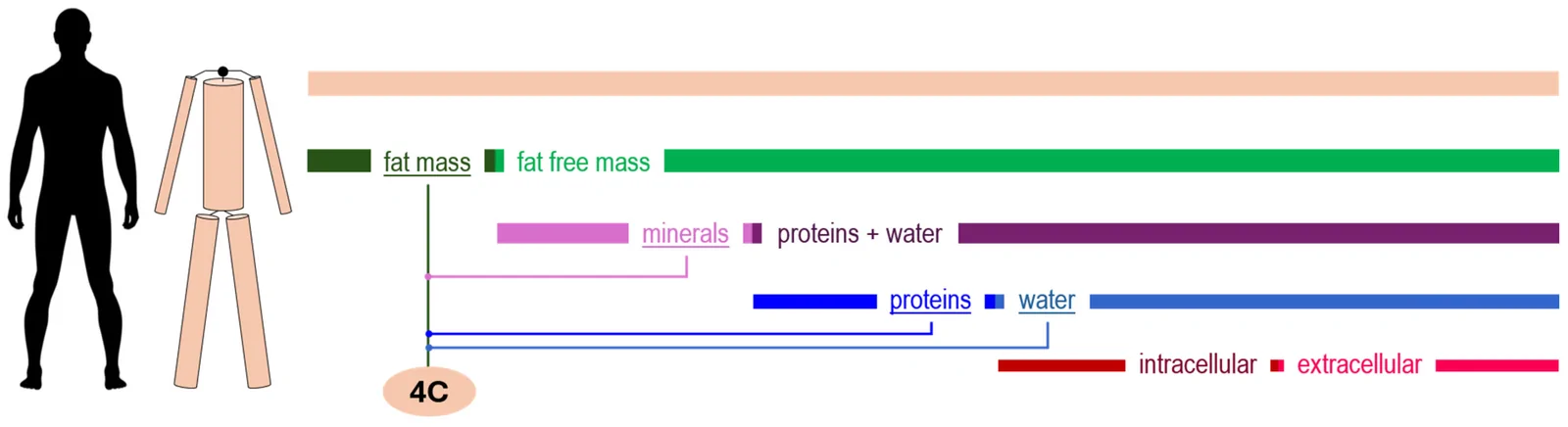
Representative body composition compartmental model in SBIA. Different manufacturers employ different compartmental models and prediction algorithms to estimate body composition.

Bioimpedance does not measure body composition directly. Instead, it determines the electrical properties of biological tissues. From these properties, several physiologically meaningful compartments can be estimated through validated prediction models. Figure 3 summarizes the hierarchical organization of human body composition. It also illustrates the principal compartments that can be estimated using SBIA.

At the highest level, total body weight can be divided into FM and FFM. FM comprises all lipid-containing tissues, whereas FFM includes all metabolically active tissues, including skeletal muscle, internal organs, bone, connective tissues, and TBW. This compartment can be further subdivided into mineral mass and soft lean tissues. A total of 95% of minerals are contained in bones, and, in fact, the mineral compartment is usually referred to as Bone Mineral Content (BMC). The combination of FM, BMC, proteins and water is usually referred to as the four-compartment (4C) model.

Cortical bone exhibits high electrical resistivity because of its low water content and highly mineralized structure. However, the contribution of bone to the measured impedance is limited, as is that of the tissues enclosed by the cortical bone. This is because bone represents a relatively small fraction of the conductive volume. Conversely, skeletal muscles and visceral organs contain large amounts of intracellular and extracellular fluids and therefore dominate the electrical conduction pathways. As a result, bioimpedance measurements are particularly sensitive to changes in lean soft tissue.

The lean soft-tissue compartment can be further separated into proteins and TBW. Structural proteins constitute the solid component of muscle and organ tissues, whereas TBW forms the conductive medium through which electrical current flows.

**Figure 4:**
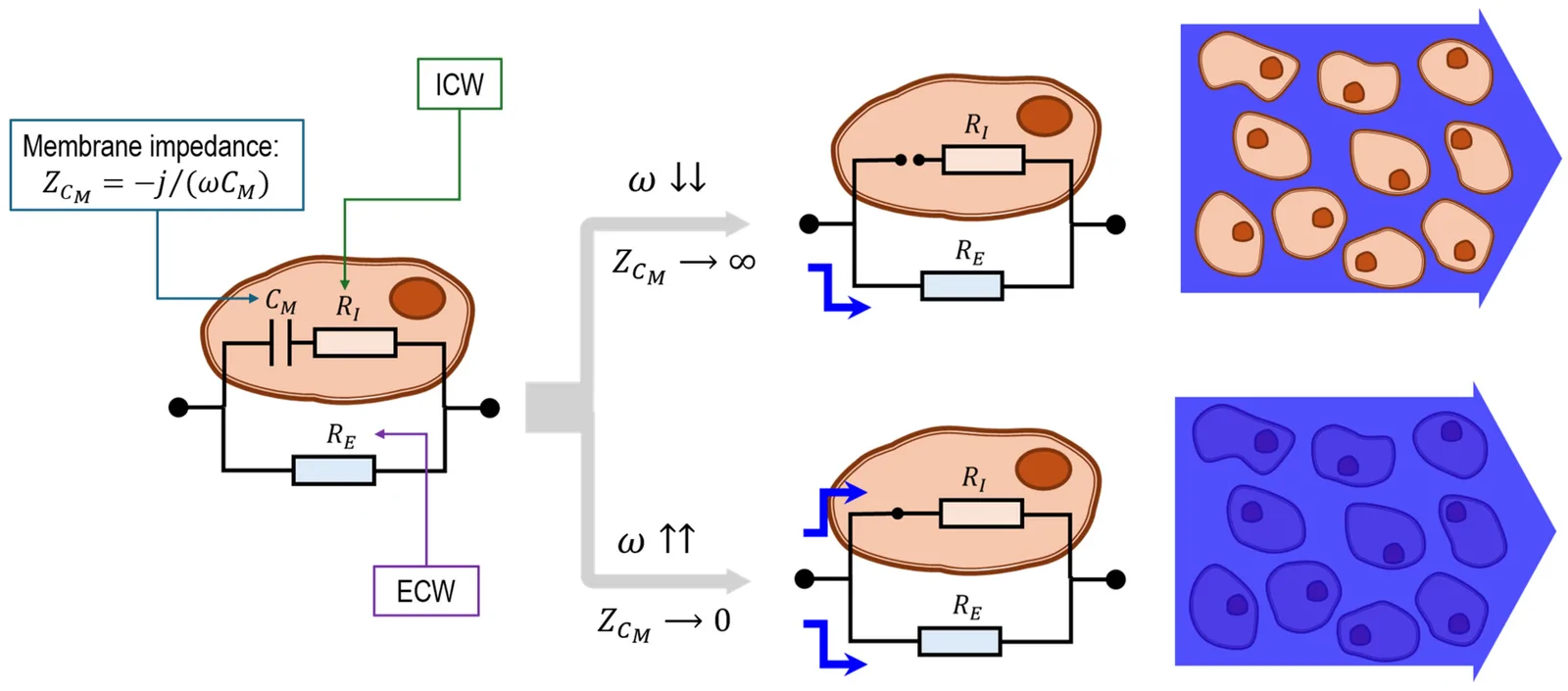
Single-cell-equivalent circuit and behavior at very low and very high frequencies.

Both MF-BIA and BIS enable the independent estimation of ECW, ICW, and TBW by exploiting the frequency dependence of tissue impedance. Figure 4 depicts a simplified equivalent circuit of a cell together with the corresponding current pathways at low and high excitation frequencies.

The selective sensitivity of low frequencies to ECW and of high frequencies to TBW provides the physical foundation for estimating ICW and for assessing changes in hydration, nutritional status, and cellular integrity. Each biological cell can be represented by an equivalent electrical circuit consisting of an intracellular resistance (*R*_I_) surrounded by a cell membrane. Electrically, the membrane behaves predominantly as a capacitor (*C*_M_) because its phospholipid bilayer acts as an excellent dielectric separating two conductive electrolyte solutions. This makes the membrane impedance strongly frequency-dependent, decreasing as the frequency increases.

At low excitation frequencies, the membrane capacitive reactance is very large. This effectively prevents alternating current from entering the cells. The current follows the path of least impedance and flows almost exclusively through ECW, bypassing the intracellular compartment. As illustrated in the upper part of Figure 4, current streamlines deviate around the cells because membranes behave as insulating barriers. Consequently, the measured impedance primarily reflects extracellular electrical conductivity. It is therefore highly correlated with ECW volume.

As the excitation frequency increases, the membrane reactance progressively decreases. At high frequencies, the capacitive impedance becomes sufficiently small for the current to cross the cell membranes and enter the intracellular space. The membranes no longer constitute a significant obstacle to current flow, allowing both the extracellular and intracellular electrolyte solutions to participate in electrical conduction. As illustrated in the lower part of Figure 4, the current traverses the cells rather than circumventing them, increasing the effective conductive volume. ICW can be obtained simply as

(6) ICW=TBW−ECW

### 2.4. Frequency Dependence and Tissue Electrical Properties

The frequency-dependent dielectrical behavior of biological tissues dictates how different body constituents can be identified and quantified across the spectrum. The resistance of a biological tissue is fundamentally determined by its electrical conductivity *σ*, an intrinsic physical property that quantifies how easily a material allows free ions to move under an applied electric field. Resistance is inversely proportional to *σ*, and mediated by the geometry of the tissue. For the simple geometry of a cylinder of length *L* and cross-sectional area *A*,

(7) R=L/(σA)

The electrolytic solutions making up the intracellular and extracellular fluids provide a high density of mobile ions that drive this conductivity, allowing the medium to dissipate electrical energy as heat.

Tissue reactance is directly connected to dielectric permittivity $\varepsilon_{r}^{\prime }$, a physical property that measures a material’s capacity to polarize and store energy within an electric field. Cell membranes—formed by an insulating lipid bilayer sandwiched between conductive media—act as biological capacitors whose capacitance is proportional to their permittivity. When an alternating current is applied, this structural arrangement temporarily stores charge and induces a phase shift in the voltage signal, which macroscopically translates into capacitive reactance.

Within the frequency range typically employed in bioimpedance measurements, the impedance spectrum is well-described by the classical Cole–Cole model:

(8) $$Z\left(\omega \right)=R_{\infty }+\frac{R_{0}-R_{\infty }}{1+{\left(j\omega \tau \right)}^{\alpha }}$$

where $R_{0}$ is the resistance at zero frequency, corresponding to current flowing exclusively through the extracellular fluid, $R_{\infty }$ is the high frequency resistance, at which current is assumed to flow through both extracellular and intracellular compartments, τ is the characteristic relaxation time, and α (0<α≤1) describes the broadening of the dielectric dispersion.

In whole-body bioimpedance measurements, the impedance locus follows a depressed semicircular arc rather than the ideal semicircle predicted by a single-time-constant RC circuit. This indicates that the frequency-dependent electrical behavior of biological tissues is governed by a distributed relaxation process. As the excitation frequency increases, the measured impedance moves along the Cole arc from right to left.

**Figure 5:**
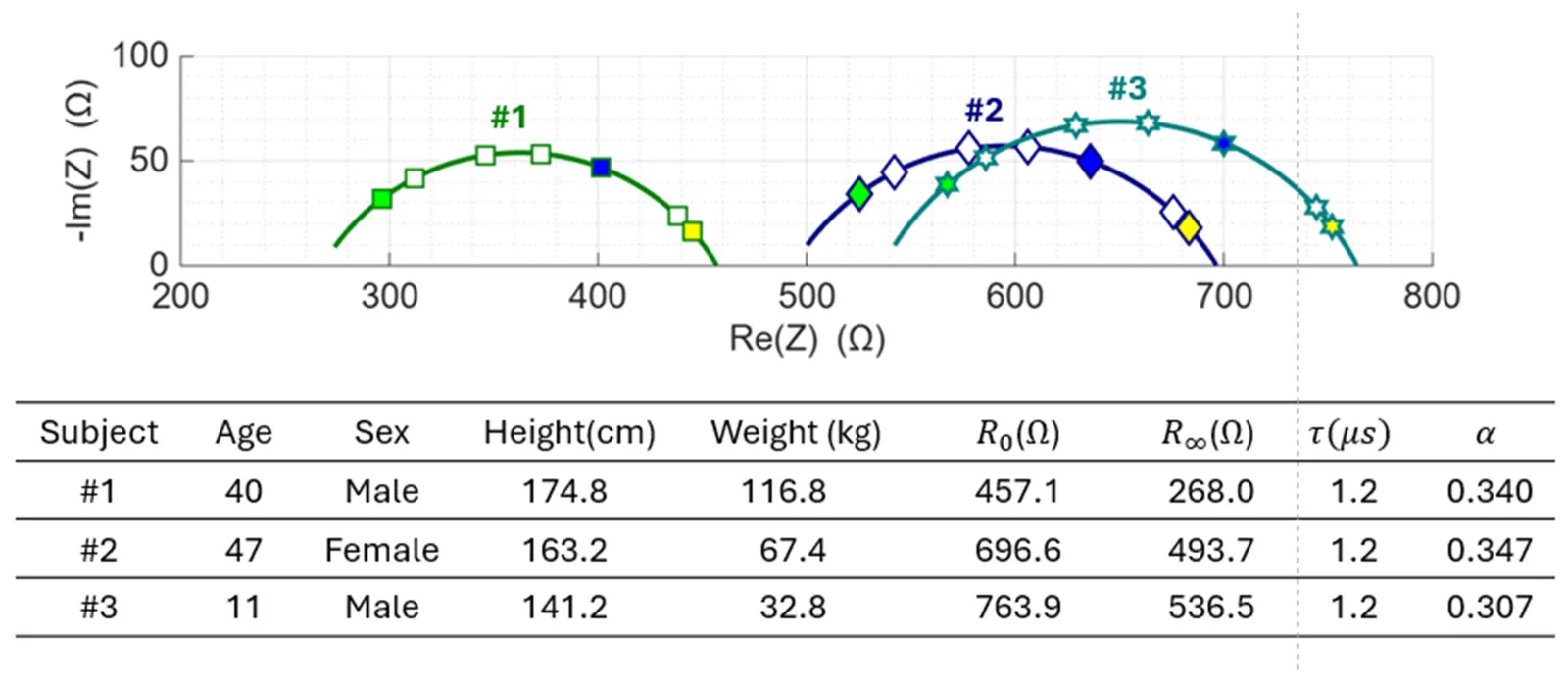
Cole–Cole plots of complex impedance for three individuals from the multifrequency bioelectrical impedance records of the NHANES 1999–2000 cycle, identified by index only. Solid curves are simulations of the Cole model of Equation (8), evaluated from 10 Hz to 10 MHz, and therefore extending beyond the measured range. Markers with white fill denote measurements at 10, 100, 200 and 500 kHz. Filled markers denote 5 kHz (yellow), 50 kHz (blue) and 1 MHz (green). The table lists age, sex, height and weight of each individual, together with the fitted values of *R*_0_, *R*_∞_, *τ* and *α*.

Figure 5 illustrates this behavior for three individuals from the multifrequency bioimpedance records of the NHANES 1999–2000 cycle, a public and de-identified dataset [32]. No measurement was performed for the present work. The three loci differ mainly in their position along the real axis. Both *R*_0_ and *R*_∞_ increase as body size decreases, which is consistent with the dependence of whole-body resistance on the geometry of the conductive path. The diameter of the arc, proportional to *R*_0_ − *R*_∞_, reflects the progressive recruitment of ICW as the frequency increases. Its height is related to the reactance and therefore to the capacitive behavior of cell membranes.

Two parameters vary far less than the resistances. The exponent *α* stays close to 0.32 in the three records, and the characteristic time *τ* is indistinguishable at the resolution reported here, near 1.2 μs, which corresponds to a characteristic frequency of about 130 kHz. This is not unexpected. *R*_0_ and *R*_∞_ scale with the length and cross-sectional area of the conductive volume, whereas *τ* and *α* depend on intensive properties of the tissue, namely the membrane capacitance per unit area and the resistivity of the intracellular and extracellular media. A similar relaxation time across individuals is therefore consistent with the model. The observation should not be over-interpreted. With a finite number of measured frequencies, *τ* and *α* are the least well-determined parameters of the fit, and three records say nothing about the range of variation expected in a population.

Both electrical conductivity and dielectric permittivity exhibit strong frequency-dependent variations known as dielectric dispersions. In the spectrum where commercial devices operate, this phenomenon is primarily driven by the structural polarization of cell membranes and the relaxation of proteins, causing permittivity to drop dramatically and conductivity to increase as the frequency of the applied electric field rises.

**Figure 6:**
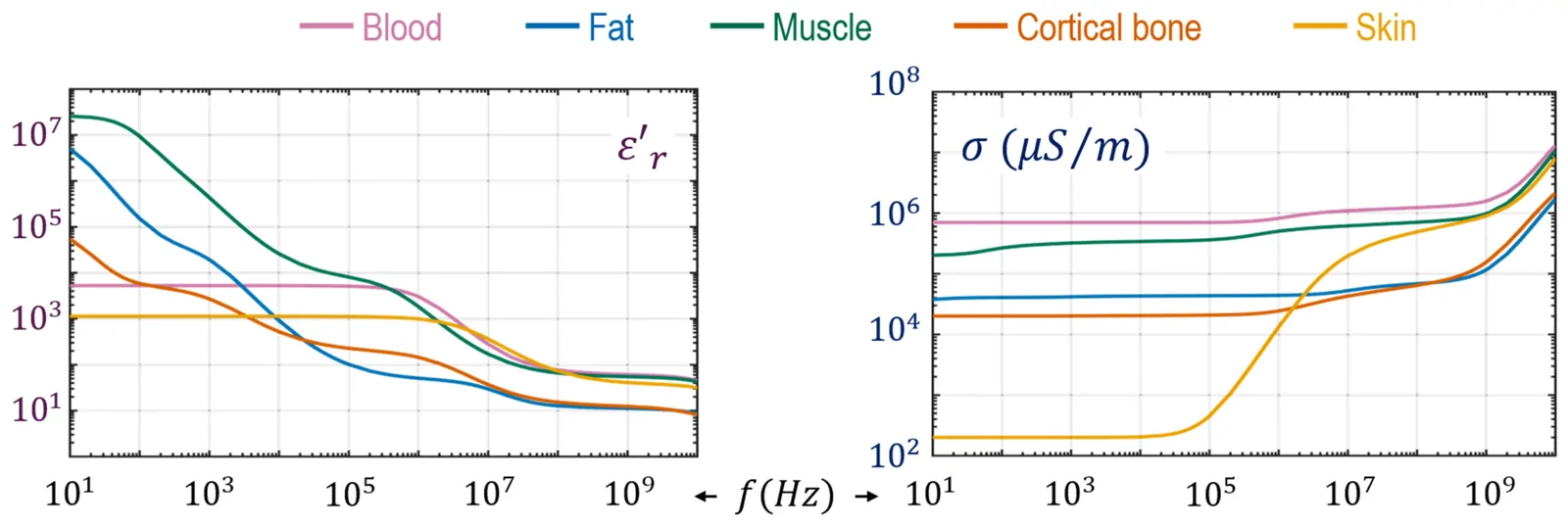
Frequency dependence of the relative dielectric permittivity (**left**) and electrical conductivity (**right**) of relevant human body constituents, according to IT’IS database [33].

Figure 6 depicts the relative permittivity and electrical conductivity of five primary body constituents as a function of the frequency, according to IT’IS database [33]. A pronounced contrast appears between highly hydrated tissues with rich extracellular fluid networks, such as muscle and blood, and poorly hydrated tissues such as adipose tissue and cortical bone. This occurs because current is restricted to extracellular pathways at low frequencies. Highly hydrated tissues manifest both elevated permittivity and conductivity, whereas fat and bone maintain significantly lower values. This wide gap in dielectric properties at low frequencies provides the necessary sensitivity to differentiate between FFM and FM, making low-frequency measurements very useful for characterizing basic tissue composition.

As the measurement frequency increases into the megahertz and gigahertz ranges, the dielectric properties undergo significant transitions known as beta and gamma dispersions. During beta dispersion, which occurs primarily in the radiofrequency range, the capacitive reactance of the cell membranes decreases to the point where the lipid bilayers become electrically transparent. While this enables the measurement of TBW, it simultaneously reduces the structural contrast between different tissue types. At the gigahertz range gamma dispersion takes over, dominated by the dielectric relaxation of free water molecules. In this domain, the conductivity of all tissues rises sharply and converges toward similar orders of magnitude, which drastically diminishes the sensitivity required to isolate or differentiate specific body constituents based purely on their electrical properties.

The electrical behavior of skin introduces a challenge in SBIA instrumentation. At low frequencies, skin exhibits very low values of σ, being several orders of magnitude lower than that of blood or muscle. This low conductivity is primarily a characteristic of the stratum corneum, the outermost layer of dead skin cells that acts as a robust insulating barrier.

In addition, the electrical conductivity of the cortical bone remains notably low at frequencies below 1 GHz, implying that the dense cortical shell restricts current penetration into deeper tissues. Consequently, physiological changes within the bone marrow are unlikely to be detected using BIA.

## 3. From Bioimpedance to Body Composition

### 3.1. BIA Prediction Equations

BIA Prediction equations (BPEs) remain largely empirical. Most models combine impedance variables with anthropometric parameters calibrated for specific devices and populations. A total of 106 BPEs derived from 64 studies involving more than 40,000 participants have recently been compiled and analyzed [34]. Likewise, Kampo et al. reviewed more specifically multifrequency BPEs published between 2000 and 2025 [35]. Population-specific equations continue to be developed, such as an FFM equation derived and validated in Greek Caucasian adults [36].

For most manufacturers, the BPEs implemented in commercial bioimpedance analyzers are not publicly disclosed. These equations are typically developed from reference body composition techniques applied to extensive population studies, conducted by the manufacturer itself. Consequently, body composition estimates obtained with different commercial instruments are not interchangeable. Moreover, estimates from a commercially available bioimpedance analyzer can still be globally refined using DXA-derived correction factors, even when cohorts comprise more than 900 individuals [37].

Table 2 presents three representative BPEs proposed by Lukaski et al. [38], Kyle et al. [39], and Hughes et al. [40], all of which showed an excellent agreement with established reference methods. We use the term ALSM (appendicular lean soft mass) to refer to the variable estimated by the equation of Kyle et al., although the original publication uses ASMM (appendicular skeletal muscle mass). Both terms refer to closely related physiological compartments and are frequently used interchangeably in the literature. ALSM, or the equivalent of appendicular lean soft tissue (ALST), is now preferred in DXA-based research because DXA measures this component directly.

Each equation can be decomposed into two additive components: an electrical term, which depends on the bioimpedance, and a measurement-independent factor (MIF). MIF is introduced in this work to represent the contribution of all regression terms that are independent of the measurement. The decomposition is an exact algebraic rearrangement of the published equation rather than a statistical estimate, and it applies only where the impedance and the anthropometric terms enter additively. The coefficient of determination quantifies the proportion of the variance in the reference body composition variable explained by the equation. Values closer to unity indicate a better fit. The standard error of estimation (SEE) represents the typical prediction error of the regression model with respect to the reference method.

**Figure 7:**
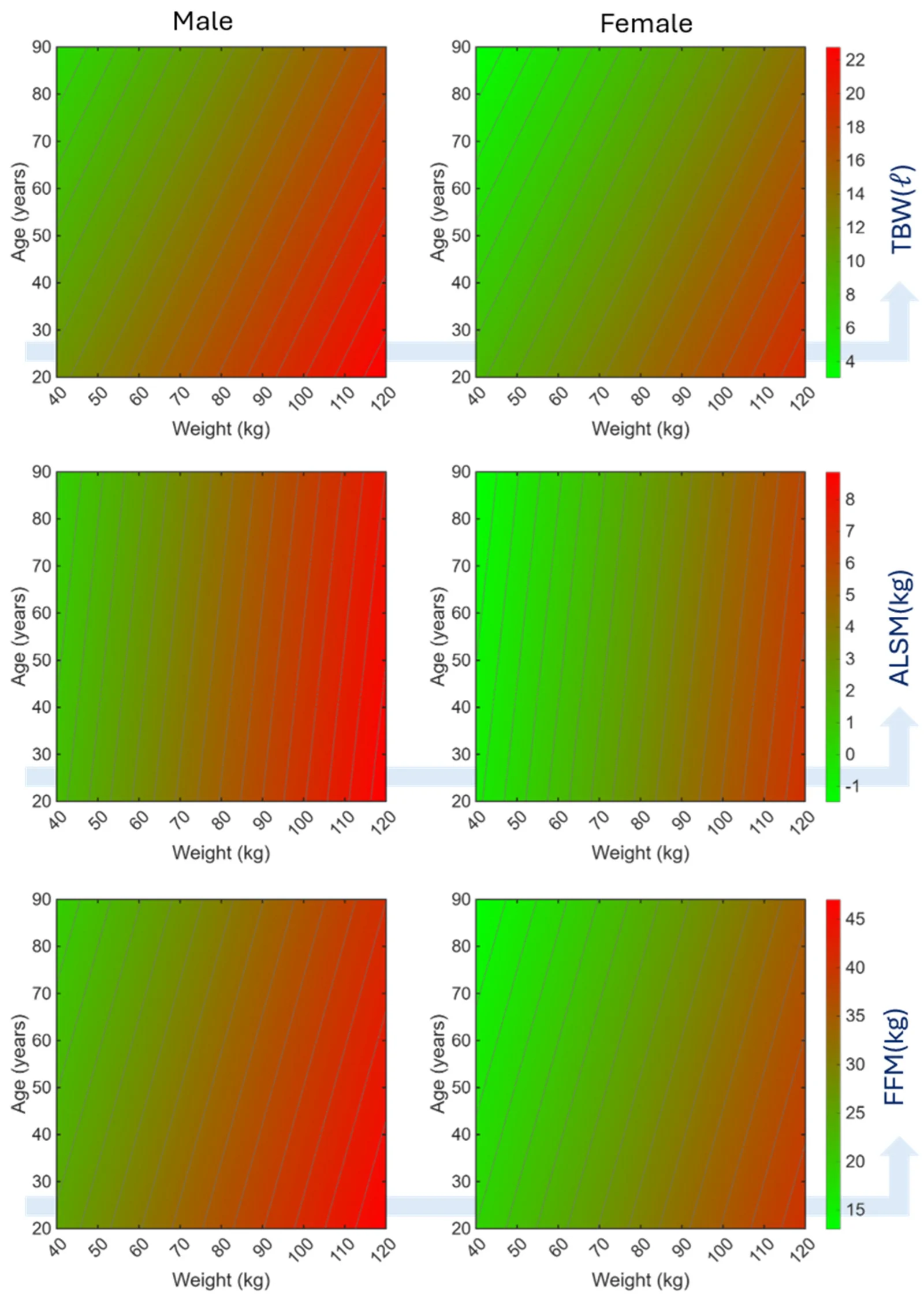
MIF contributions to three prediction equations: TBW according to Lukaski et al. equation, ALSM according to Kyle et al. equation, and FFM according to Hughes et al. equation. Gray contour lines represent equal predicted values.

As illustrated in Figure 7, MIF is primarily determined by the subject’s anthropometric characteristics, namely body weight, age, sex, and regression constants. Among these variables, body weight exerts the strongest influence, whereas age introduces a comparatively smaller negative correction. The effect of sex is reflected as an approximately constant offset between the male and female prediction surfaces, without modifying their overall shape or gradient.

MIF varies over a wide range within the physiological intervals considered. For all three prediction equations, the largest values are obtained in young individuals with high body weight, whereas the lowest values correspond to elderly subjects with low body weight. The nearly parallel contour lines indicate that the anthropometric contribution changes almost linearly throughout the entire range of age and body weight, reflecting the linear structure of the underlying regression models.

This example illustrates that the MIF can contribute as much as, or even more than, the electrical measurement itself. Consequently, a substantial proportion of the estimated body composition may already be determined before any bioimpedance data are incorporated into the prediction model. However, the opposite may also occur: for certain weight–age combinations, the ALSM equation of Kyle et al. predicts that the MIF cancels out, so the resulting ALSM depends solely on the measurement.

MIF is not a measure of statistical importance, and it is not a share of explained variance. A term may dominate the predicted value while accounting for little of the variance explained across a cohort. The magnitude of the MIF is furthermore specific to each equation and to the anthropometric range considered, so the values illustrated in Figure 7 should not be extrapolated to other equations or to populations outside that range.

### 3.2. DXA-Based Calibration and Validation of BPEs

DXA plays a major role in the validation of BPEs. Although originally introduced for the assessment of bone mineral density [41], advances in detector technology and image processing have transformed DXA into one of the most widely accepted methods for whole-body composition assessment [42,43].

The physical principles underlying DXA have been comprehensively described by Pietrobelli et al. [44]. The technique is based on irradiating the body with two X-ray photon energies. Because bone mineral, lean soft tissue, and adipose tissue exhibit different X-ray attenuation coefficients, the simultaneous acquisition of low- and high-energy images allows each pixel to be decomposed into its constituent tissue components. By integrating information from all image pixels, DXA provides estimates of whole-body and regional body composition with high spatial resolution and excellent reproducibility.

BPEs can be calibrated against the 4C model. The 4C model is generally regarded as the criterion method for in vivo body composition assessment because it minimizes the simplifying assumptions required to estimate body composition. FM, TBW, BMC and proteins are quantified using independent reference techniques, typically isotope dilution for TBW, air-displacement plethysmography or hydrodensitometry for body volume, and DXA for BMC. FM and proteins are subsequently calculated with multicompartment equations [45].

Despite its superior accuracy, the 4C model is impractical for the development of BPEs because it requires multiple independent measurements and time-consuming protocols. In contrast, DXA provides simultaneous estimates of all body compartments in a single examination with high precision and low cost. For this reason, commercial BPEs can be calibrated directly against DXA.

DXA depends on expensive instrumentation and entails regular calibration, radiation safety procedures, and trained operators. Although the radiation dose is relatively low—typically between 1 and 10 μSv for a whole-body scan—it nevertheless limits the frequency of repeated measurements, particularly in children, pregnant women, and longitudinal studies requiring frequent follow-up. A complete examination generally requires 5–15 min, substantially longer than a conventional BIA measurement. Furthermore, DXA systems are neither portable nor suitable for bedside or home monitoring.

Whole-body DXA measurements typically exhibit coefficients of variation below 1% for total body mass and approximately 1–2% for lean and fat soft tissues [46]. Nevertheless, Park et al. showed that body composition estimates obtained with different DXA systems are not always directly interchangeable, particularly for FM estimation [47]. More recently, several studies have demonstrated the feasibility of cross-calibrating measurements obtained with different DXA platforms [48,49]. Caution must be taken when comparing DXA results from different manufacturers, since terms like lean mass or ALST are used inconsistently [50].

Another limitation concerns the estimation of visceral adipose tissue (VAT). Although modern DXA systems incorporate dedicated algorithms for VAT quantification, these estimates are derived indirectly from two-dimensional projection images rather than true tomographic data. Kaul et al. [51] reported good agreement between DXA-derived VAT estimates and CT, although systematic differences persisted, particularly in individuals with severe obesity or atypical fat distribution. Likewise, Borga et al. identified CT and MRI as the preferred techniques for accurately quantifying VAT, diffuse fat infiltration, and individual muscle volume [52]. More recently, Mancilha et al. found that DXA systematically overestimates VAT compared to MRI [53].

DXA is recommended for assessing FM across many clinical conditions; however, its validity for evaluating FFM in clinical populations remains uncertain [54]. In addition, Basty et al. recently reported that DXA is less accurate than MRI for FFM assessment, particularly in longitudinal studies [55]. Several studies have evaluated the agreement between BIA and DXA. Achamrah et al. compared FM and FFM estimated by both techniques and reported good agreement, particularly in individuals with a body mass index (BMI) between 16 and 18.5 kg/m^2^. However, for BMI values above 18.5 and below 40 kg/m^2^, BIA overestimated FFM by 3.38–8.28 kg and underestimated FM by 2.51–5.67 kg relative to DXA [56]. Similarly, Farias et al. demonstrated that simple correction equations can improve the agreement between DXA measurements and commercially available single-frequency leg-to-leg BIA devices [57]. More recently, Zaplatosch et al. assessed the validity of body composition estimates obtained with a multifrequency BIA device in women [58].

FM is generally not predicted directly by BIA but obtained as the difference between body weight and FFM, so the entire error of the FFM estimate is transferred to FM. FFM, in turn, is reached either from TBW, under the assumption that the hydration fraction of FFM remains constant at approximately 73.2%, or by direct regression against DXA in a particular cohort. Both routes are vulnerable in the same direction, because the constant-hydration assumption and the cohorts used for calibration are least representative at the extremes of adiposity. The magnitude of the resulting uncertainty is not negligible: the SEE of the FFM equation was previously shown in Table 2: it is close to 7% of the FFM of an adult of average build, and it corresponds precisely to the equation derived in the cohort with the highest median BMI, 31 kg/m^2^. Accuracy therefore degrades as adiposity increases, that is, in precisely the population in which body composition is most frequently assessed. This is also the regime in which descriptors that require no regression step offer the clearest advantage: BIVA has been reported as particularly valuable in severe obesity, where conventional equations often provide inaccurate estimates [25].

Commercial analyzers also report parameters whose relationship to the electrical measurement is considerably more remote. VAT and BMC are provided routinely, and BMC obtained with a commercial analyzer has been used as a primary endpoint in recent clinical work [59], yet neither is a quantity that impedance can resolve. Cortical bone is highly resistive and occupies a small fraction of the conductive volume, so a BMC output is almost entirely a product of the regression model rather than of the measurement. VAT is not a distinct electrical compartment either. Any reference used to calibrate such an estimate also carries its own systematic error, as illustrated by the discrepancies between DXA, CT and MRI described above. The practice of the recent literature is consistent with this. In the only primary study of the present corpus in which VAT enters the analysis, bioimpedance supplied body fat percentage alone and VAT was obtained by other means [60]. Likewise, methodological reviews assign visceral fat and bone mass to imaging and cite bioimpedance as a low-cost but imprecise alternative [61]. The standing of these two outputs is therefore best described as uncharacterized rather than quantified: the assessments available are qualitative.

### 3.3. Boundary Limitations Across the Frequency Spectrum

The frequency dependence of tissue impedance provides valuable information on the physiological parameters estimated by bioimpedance analysis. In practice, however, the usable frequency range is constrained by physical phenomena and instrumentation-related parasitics that progressively degrade measurement accuracy.

Table 3 summarizes the operating frequencies of representative commercial bioimpedance analyzers. Most of them perform measurements between approximately 1–5 kHz and 1 MHz, with only a few extending into the low-megahertz range.

The availability of bioimpedance measurements at very low frequencies would improve the characterization of current flow through the extracellular compartment and, consequently, provide a more reliable estimation of ECW. Why, then, do commercial bioimpedance analyzers seldom operate below approximately 1 kHz? The answer lies in the increasingly dominant contribution of electrode polarization (EP).

EP is most commonly associated with electrodes immersed in liquid electrolytes [62,63], but the same electrochemical processes also occur at the interface between a metallic electrode and biological tissue [64]. In bioimpedance measurements, EP originates at the electrode–skin interface. Whereas electrical conduction in metals is mediated by electrons, biological tissues conduct current through the movement of ions. Because these charge carriers cannot be exchanged directly across the interface, ions accumulate near the electrode’s surface, forming an electrical double layer that behaves as a capacitive element. As the excitation frequency decreases, the impedance of this interfacial capacitance increases rapidly and may eventually become several times larger than the impedance of the body segment being measured. Under these conditions, the measured impedance increasingly reflects the electrode–skin interface rather than the tissue itself.

EP therefore introduces a systematic error into the measurement. Why not simply remove this contribution through calibration or mathematical compensation? Several equivalent circuit models have been proposed using a constant phase element aiming at compensating for EP effects [65,66]. These approaches can improve measurement accuracy under controlled laboratory conditions. However, their effectiveness is limited in routine clinical practice. EP depends on numerous factors, including electrode material, skin preparation, contact pressure, hydration, and subject-to-subject variability. Consequently, minimizing EP at its source remains the preferred strategy.

The tetrapolar electrode configuration adopted in most commercial bioimpedance analyzers substantially reduces the influence of EP by separating the current-injecting and voltage-sensing electrodes, although it cannot eliminate the effect completely. A complementary approach is the use of Ag/AgCl electrodes, which remain the reference standard for bioelectrical measurements owing to their highly reversible electrochemical behavior based on the Ag/AgCl/Cl^−^ equilibrium [67]. This reversible reaction provides a stable half-cell potential and a considerably lower polarization impedance than conventional metallic electrodes. However, their performance relies on a chloride-containing hydrogel to maintain a stable electrode–skin interface, making them susceptible to gel dehydration, skin irritation, and reduced long-term stability.

At the opposite end of the spectrum, extending bioimpedance measurements beyond the conventional frequency range could provide additional physiological information by probing tissue dispersion phenomena. Moreover, because the impedance of the electrode–skin interface decreases markedly with increasing frequency, EP becomes progressively less important. However, these benefits are accompanied by new challenges.

As the operating frequency approaches the megahertz range, parasitic effects associated with the measurement hardware become increasingly significant [68]. Stray capacitances in cables, connectors, electrode leads and switching circuits introduce amplitude and phase errors, while mutual inductive coupling and electromagnetic interference become more difficult to control.

In addition, when the physical dimensions of the measurement system are no longer negligible compared to the effective signal wavelength, the lumped-element approximation ceases to be valid and distributed transmission-line effects must be considered. These phenomena ultimately limit the practical upper frequency that can be used for accurate in vivo bioimpedance measurements.

## 4. Latest Advances of Interest for Clinical Nutrition

### 4.1. Electrode Technologies and Bioimpedance Wearables (BWs)

In clinical nutrition, bioimpedance analysis is increasingly used not only to estimate body composition but also to monitor changes in hydration, muscle mass and nutritional status during disease progression and nutritional interventions. As nutritional care shifts toward longitudinal patient follow-up, there is an increasing demand for reliable bioimpedance devices. Advances in electrode technologies, flexible electronics and wearable devices have become key drivers. Electrode technology is a critical component of BWs because the electrode–skin interface largely determines measurement reproducibility and long-term stability. It has become an active research field not only within the bioimpedance community but also in related disciplines such as biopotential monitoring [69] and human–machine interaction [70], which face similar challenges regarding electrode–skin impedance and contact reliability.

The transition from conventional Ag/AgCl gel electrodes toward dry, flexible and printable electrodes has become one of the main research directions in biopotential wearables and BWs. Materials under investigation include noble metals, conductive polymers, graphene and conductive textiles [71]. Kusche et al. [72] evaluated dry, miniaturized electrodes (177 mm^2^ surface area) fabricated from various materials. While conventional Ag/AgCl electrodes exhibited the lowest interface impedance, metal (gold-plated and stainless steel) and carbon rubber electrodes achieved comparable performance, demonstrating their suitability for bioimpedance measurements. Nevertheless, maintaining a stable, low-impedance interface during prolonged use and under dynamic conditions remains the main challenge for wearable bioimpedance devices.

Recent advances in printed electronics have further expanded the capabilities of BWs. Levit et al. [73] developed flexible skin electrodes fabricated by combining screen-printed carbon with inkjet-printed PEDOT:PSS. Soft, conformable electrode arrays were achieved with low contact impedance and long-term stability.

The electrodes enabled reliable four-electrode bioimpedance measurements over several hours, including the continuous monitoring of arterial pulsations and during simultaneous muscle activity. These advances support future BWs for repeated assessment of body composition and hydration in free-living individuals, older adults and patients receiving nutritional support.

Despite these advances, continuous monitoring under daily living conditions imposes stringent requirements on electrode design. Sweat accumulation, changes in contact pressure and body motion influence the electrical properties of the electrode–skin interface, leading to motion artifacts and measurement variability. Machine-learning approaches are increasingly being employed to suppress motion artifacts, extract clinically relevant physiological features and integrate bioimpedance measurements with complementary sensors. This improves measurement robustness and enables reliable physiological monitoring during daily activities [74]. In addition, efficient low-power signal-processing algorithms have become an essential component of BWs [75]. Groenendaal et al. [76] highlighted the potential of BWs for chronic disease management, emphasizing continuous home monitoring with low-cost, low-power devices. In clinical nutrition, BWs could facilitate the longitudinal assessment of hydration, lean tissue preservation and nutritional recovery in patients with chronic diseases.

Wearable EIT (WEIT) devices provide spatially resolved information on tissue composition. Chen et al. [77] identified WEIT as an emerging technology for continuous physiological monitoring, highlighting the integration of flexible electronics, textile-based sensors and intelligent signal processing. These systems enable real-time monitoring of muscle activity and soft-tissue composition, supporting the longitudinal evaluation of muscle health outside conventional clinical settings.

In clinical nutrition, the continuous monitoring of muscle quality, body composition and functional decline could facilitate the early detection of malnutrition and sarcopenia, while improving the follow-up of patients receiving nutritional interventions. WEIT has also shown promise for the non-invasive evaluation of hepatic steatosis. Luo et al. [78] demonstrated that a portable WEIT system can estimate liver fat content, with measurements correlating with MRI-derived liver fat and discriminating between different degrees of hepatic fat accumulation. Although further clinical validation is required, these findings suggest a potential role for BWs in the longitudinal monitoring of metabolic dysfunction-associated steatotic liver disease during nutritional interventions.

### 4.2. Bioimpedance Imaging

Whole-body BIA condenses the electrical behavior of the entire organism into a few global descriptors, which is insensitive to the regional nature of most nutrition-related alterations. Muscle wasting in sarcopenia and cachexia progresses unevenly across limbs and trunk. Fluid overload accumulates preferentially in dependent regions, and refeeding-associated fluid shifts are inherently local. Imaging-based bioimpedance resolves where conductivity changes occur rather than only how large they are, while remaining non-invasive, radiation-free, inexpensive, and repeatable at the bedside. These properties suit the longitudinal monitoring of nutritional interventions, where the relevant signal is a change in regional muscle quality or fluid distribution rather than a single cross-sectional estimate. EIT and Electrical Impedance Myography (EIM) extend conventional bioimpedance analysis by providing regional information about tissue function rather than whole-body electrical properties.

EIT reconstructs the internal conductivity distribution of biological tissues from voltage measurements acquired using multiple surface electrodes. Unlike conventional anatomical imaging modalities, EIT provides continuous bedside monitoring with high temporal resolution, making it particularly suitable for functional assessment rather than structural diagnosis. EIT does not provide detailed anatomical information but continuously visualizes functional changes in tissue conductivity.

The growing clinical adoption of EIT was further supported by the international consensus statement of the TREND group [79], which established standardized examination protocols, unified terminology, data analysis methods, and recommendations for the clinical interpretation of thoracic EIT. Youssef Baby et al. [80] summarized the evolution of both EIT and EIM technologies, highlighting the growing role of digital signal processing, machine learning, and integrated electronic systems in improving image quality and diagnostic performance.

Recent progress in chest EIT has been reviewed by Frerichs et al. [81], who describe its increasing use for the bedside monitoring of pulmonary perfusion and patient-specific optimization of mechanical ventilation. Their review illustrates how technological advances and improved reconstruction algorithms have accelerated the clinical adoption of EIT in intensive care medicine, particularly for patients requiring mechanical ventilation.

Beyond respiratory monitoring, EIT is progressively expanding toward cardiovascular assessment, neurocritical care and gastrointestinal monitoring [80]. Although limitations in spatial resolution remain, ongoing improvements in reconstruction algorithms and hardware design continue to broaden its diagnostic potential.

EIM focuses on the localized assessment of skeletal muscle, providing quantitative information on muscle composition, membrane integrity and neuromuscular function. Sonbas–Cobb et al. [82] recently demonstrated that combining multifrequency EIM with machine-learning algorithms improves the screening of neuromuscular disorders compared to conventional impedance metrics alone. Their results illustrate the growing integration of artificial intelligence into impedance-based diagnostic systems and support the further evaluation of EIM as a candidate objective biomarker for neuromuscular disease detection.

Current research is also extending impedance-based muscle assessment beyond clinical diagnosis toward rehabilitation engineering and human–machine interaction. Park et al. [70] describe how EIM can be combined with biopotential recordings to improve prosthetic control, rehabilitation monitoring and wearable biomechanical interfaces. Hybrid impedance–EMG sensing decodes movement intention more robustly than EMG alone, particularly during dynamic contractions and when conventional EMG signals become unstable. This illustrates the increasing convergence between bioimpedance imaging, intelligent sensing, and digital healthcare.

Bioimpedance-based imaging technologies grapple with intrinsic physical limitations. Spatial resolution remains notably low compared to structural modalities such as CT or MRI. Furthermore, image fidelity strictly depends on the exact placement of electrodes and is heavily affected by the anatomical shape variability of each patient’s chest or limbs.

### 4.3. Toward Standardized Bioelectrical Biomarkers

Despite its widespread use for body composition assessment in healthy populations, BIA has yet to achieve broad clinical acceptance. Reference values for BIA-derived parameters have been proposed to aid clinical interpretation, such as those of Barbosa et al. for PhA [83]. However, in its 2020 evidence-based clinical guideline, the American Society for Parenteral and Enteral Nutrition (ASPEN) reached a different conclusion. In its view, the available evidence was insufficient to recommend BIA as a reference method for body composition assessment in hospitalized patients or in individuals with acute or chronic diseases [54]. This conclusion was based on four main limitations: (i) limited validation of BIA methods in clinical populations, (ii) substantial variability among commercial devices, (iii) the influence of disease-related factors like inflammation, and (iv) insufficient high-quality evidence. This last point refers to the lack of adequately powered studies using appropriate reference methods and clinically relevant outcomes. ASPEN also set priorities for future research: validating bioimpedance methods in clinical populations; standardizing terminology and measurement protocols. Further priorities were investigating raw bioelectrical variables as clinically meaningful biomarkers. It also called for establishing disease-specific reference values and diagnostic cutoff points, and adopting more rigorous study designs with appropriate reference methods.

Interest in raw bioelectrical variables has a long clinical history, including early applications of PhA in nutritional and oncological assessment during the 1990s. Since 2020, the field has received renewed attention, centered on standardization, reference values and outcome-based validation. PhA and BIVA emerged as the most promising candidates for clinical implementation.

Oncology illustrates this trajectory. Early studies used BIA to assess malnutrition and body cell mass in patients with cancer, and to monitor body composition during nutritional support [84,85]. The introduction of BIVA in 1994 allowed the impedance vector to be interpreted directly [23], and in 2000 patients with lung cancer were shown to occupy a distinct vector position relative to matched healthy controls [86]. By 2004, PhA had been reported as a prognostic indicator in advanced pancreatic cancer [87]. Outcome-based validation against survival developed mainly from 2020 onward.

Unlike conventional estimates of FM, FFM or TBW, PhA is a direct electrical parameter obtained from the relationship between resistance and reactance, without relying on regression equations or body composition models. Consequently, it is less affected by the methodological variability associated with proprietary algorithms and provides information that more directly reflects cellular integrity, hydration status and BCM. Its absolute value nevertheless remains dependent on instrumentation, electrode configuration and measurement conditions.

Likewise, BIVA enables the interpretation of the measured impedance vector itself, avoiding the uncertainties associated with predictive equations while providing an integrated assessment of hydration and soft-tissue characteristics.

Large-scale systematic reviews have since compiled age- and sex-specific reference values for PhA, representing an important step toward clinical standardization [88]. At the same time, comprehensive reviews highlighted the complementary roles of PhA and BIVA for assessing muscle quality, hydration status and functional performance, extending their applications well beyond conventional body composition analysis [89]. Growing evidence also demonstrated the prognostic value of PhA in different clinical settings. Prospective studies showed that low PhA predicts future functional disability in older adults [90], while systematic reviews confirmed consistently reduced PhA values in patients with cardiovascular disease, supporting its role as a prognostic biomarker for cardiovascular risk stratification [91].

In parallel, considerable efforts were devoted to establishing the physiological basis underlying these observations. Comprehensive reviews consolidated the interpretation of PhA as an indicator of cellular hydration, membrane integrity and BCM [92], while also emphasizing the need for standardized measurement protocols and clinical reference values to facilitate routine implementation [93]. Experimental studies further demonstrated that segmental PhA correlates with local muscle contractile properties in healthy individuals [94] and longitudinal investigations showed that increases in segmental PhA accompany improvements in muscle quality during post-stroke rehabilitation [95]. More recently, long-term prospective studies have shown that segmental PhA combined with the ECW/ICW ratio predicts future functional disability in older adults, introducing regional bioelectrical markers for the early detection of frailty [96]. Similarly, BIVA has proven useful for monitoring body composition changes during stroke rehabilitation, providing clinically relevant information that complements conventional anthropometric and body composition measurements [97].

The two descriptors are not, however, equally consolidated. Age- and sex-specific reference values, prognostic evidence and segmental applications have accumulated for PhA, whereas for BIVA the corresponding reference framework remains largely limited to the tolerance ellipses defined at its introduction [24]. Extending to BIVA, the reference material already available for PhA is therefore part of the standardization effort that ASPEN identified as a research priority.

A methodological refinement of particular relevance in this context is specific BIVA (sBIVA), in which resistance and reactance are normalized by an estimate of the geometry of the conductive volume instead of by height alone [98]. Because the classical vector depends strongly on body size, differences between individuals or groups may reflect body geometry rather than tissue composition. The specific normalization attenuates this dependence and improves the separation between fluid distribution and soft-tissue quality, an advantage that is most evident in obesity, where the classical vector is most affected [99]. sBIVA is conceptually aligned with the argument developed in this review: like PhA, it is obtained from the measured impedance and therefore avoids prediction-equation calibration bias, while additionally addressing the size dependence that limits the classical vector. Its interpretation nevertheless remains anchored to the tolerance ellipses of the population in which they were derived, so vector positions referred to different reference samples are not directly comparable [24].

Recent studies have also expanded the biological interpretation of PhA beyond its purely electrical definition. Mechanistic reviews have linked PhA to growth, body composition and cellular physiology across the lifespan [100]. Prospective cohort studies have demonstrated that raw bioelectrical variables, particularly PhA, can monitor body composition changes during growth without relying on prediction equations, supporting their use for longitudinal follow-up [101]. More broadly, recent reviews have summarized the growing number of clinical applications of BIA in nutrition, fluid management, oncology, cardiology and intensive care. These reviews highlight the ongoing transition from model-dependent body composition estimates toward physiologically meaningful bioelectrical biomarkers [102]. This evidence is, however, largely observational. The associations reported between raw bioelectrical parameters and clinical or nutritional outcomes are consistent across settings. However, they do not establish a causal relationship in themselves, nor do they demonstrate that management guided by these parameters improves patient outcomes.

## 5. Bibliometric Analysis of the Literature (2020–2026)

### 5.1. Article Selection and Citation Impact

The methodology and nutrition equations described in Section 1 were reused for this analysis, but without the restriction to the Article document type. Consensus documents, clinical practice guidelines and reviews are themselves objects of study in this section rather than duplicated evidence. This search was run on 28 July 2026. It was restricted to the 2020–2026 window and retrieved 5625 records, exported directly from the Web of Science interface and provided as Supplementary Materials. To identify influential studies while maintaining a manageable dataset for qualitative analysis, we retained only publications averaging at least 20 citations per year, [54,59,60,61,88,89,92,93,103,104,105,106,107,108,109,110,111,112,113,114,115,116,117,118,119,120,121,122,123,124,125,126,127,128,129,130,131,132,133,134,135,136,137,138,139,140,141,142,143,144,145,146,147,148,149,150,151,152,153,154,155,156,157,158]. The resulting dataset was reviewed to classify publication type, study design, clinical application, and the specific role played by bioimpedance within each study. Large language models were used to cross-check the summarized contents tabulated in Appendix A against the corresponding full texts.

Articles were first classified according to the “Document Type” field assigned by WoS, yielding two categories: review articles and non-review articles. Following a manual analysis of the article, it became apparent that several articles labeled as “non-review” by WoS were, in substance, consensus guidance documents, clinical practice guidelines, or systematic reviews with meta-analysis rather than studies reporting primary data. These were therefore reclassified and grouped together with the review articles for the purposes of this analysis. This process yielded a final classification of 37 review-type articles and 27 non-review articles.

Although the number of highly cited publications decreases in the most recent publication years, this pattern should not be interpreted as evidence of declining research activity. The total volume of publications continues to increase across all body composition modalities, whereas the apparent reduction in highly cited papers is consistent with citation maturation effects and the shorter time available for recently published articles to accumulate citations.

Table 4 summarizes citations per year (Cites/year) by group and by the role assigned to BIA within each document. Review-type articles accumulated more citations per year than non-review articles (Mann–Whitney U = 698.5, *p* = 0.007), and articles assigning BIA a complementary role received more citations than those treating it as the primary measurement technique (U = 322.5, *p* = 0.030). Both distributions were strongly right-skewed, driven by a small number of very highly cited consensus documents and guidelines, so the median is the more representative summary statistic. Part of this difference is a generic feature of citation behavior rather than a property of the bioimpedance literature, since review articles are cited more frequently than original research across virtually all disciplines. A selection threshold based on citations per year therefore favors synthesis over primary research by construction, and the resulting ratio of 37 review-type to 27 non-review articles should be read as reflecting both this structural effect and a genuine predominance of synthesis within the field.

### 5.2. Reviews, Guidelines, and Consensus Documents

Table A1 (Appendix A) summarizes the reviewed articles. Narrative, overview and critical reviews comprise the largest share of the literature, 59%. Articles employing formal systematic evidence synthesis, such as scoping reviews or systematic reviews with or without meta-analysis, account for 22%. Consensus statements and clinical practice guidelines constitute the remaining 19%.

This distribution reveals that the most-cited BIA literature in clinical nutrition rests predominantly on expert interpretation rather than pooled quantitative synthesis. The chronological distribution is comparatively even, indicating sustained review interest across the period.

Analysis of the articles showed that sarcopenia was the most frequently addressed clinical area within the review group. These covered diagnostic criteria and screening algorithms [103,118,123,126,127], as well as prevalence estimates in the general population and in specific settings such as dialysis [115,117,132]. They also addressed underlying molecular and pathogenic mechanisms [126,134], the use of raw bioelectrical parameters, particularly PhA, in this population [109], and the interplay with cardiovascular aging [133]. A consensus document on sarcopenic obesity outlines research priorities for this overlapping phenotype [124].

The second-largest group is methodological rather than disease-oriented, focusing on body-composition assessment itself [54,61,88,92,93,104,107,110,119,128,131]. Four of these studies are specifically devoted to PhA [88,92,93,128]. This confirms the shift toward raw bioelectrical biomarkers described above. Reviews devoted to sports and athletic populations [108,112], together with a systematic review addressing methodological aspects of BIA in athletes [119], malnutrition assessment, muscle-health knowledge-translation resources [116,120,121], and oncology [113,114,122] followed.

The remaining areas were represented by one or two articles each: obesity by two, one addressing metabolic phenotypes and biomarkers [105] and the other the adequacy of BMI-based diagnosis [125]. Chronic kidney disease [106], critical illness [111], cystic fibrosis [129], and menopause-related nutrition [130] were represented by a single article. Fluid overload and altered hydration are precisely the conditions in which prediction-equation-based estimates of body composition are least reliable. The scarce high-impact review coverage of critical illness and renal disease is therefore notable, and marks these settings as the clearest gaps in the current evidence.

A further distinction emerges from the role assigned to BIA within each document. Only about one quarter of the reviewed documents treat BIA as their primary object of study [88,92,93,108,109,111,119,122,128,131]. In the rest, it appears as a complementary technique, typically listed as one acceptable muscle-mass or FM method alongside DXA, CT, or MRI.

Raw bioelectrical descriptors are discussed in 21 of the 37 documents and BIVA in 11. Five documents identify device-, population- or equation-specific variability as a central limitation [93,107,110,116,120], so the reporting gap is flagged by the same literature that leaves it open. DXA acts as the comparator of reference in 18 entries, ahead of CT in ten and MRI in three, which frames BIA as a technique benchmarked against imaging rather than against a criterion measure of body composition. Where numerical PhA thresholds are offered, they do not converge. Reported values include 3.55–5.05° for sarcopenia screening [109], <5.58° for nutritional risk in critical illness [111], 5.95° for 5-year survival in advanced head-and-neck cancer [122], and <5.10° in men and <4.73° in women for low muscle radiodensity [128]. The only value repeated anywhere in Table A1 and Table A2, 5.95°, is derived for two unrelated endpoints, which illustrates that numerical agreement between studies can be coincidental rather than substantive.

### 5.3. Studies Reporting Primary Data

Table A2 (Appendix B) presents 27 non-review articles. By study design, cross-sectional studies were the most frequent (n = 11, 41%), followed by prospective cohort studies (n = 8, 30%), randomized controlled trials (n = 4, 15%), other observational designs (n = 2, 7%), and methodological or development studies (n = 2, 7%). One of the four randomized trials used cluster randomization at the practice level [144]. One of the eight prospective cohorts is a post hoc analysis of a previous cohort [141], and one of the cross-sectional studies is a cross-sectional analysis nested within a prospective cohort [158]. Designs capable of supporting temporal or causal inference—prospective cohorts and randomized trials—thus accounted for 44% of this group, a stronger evidentiary profile than that of the review literature, in which formal evidence synthesis represented only 22% of the articles.

Sarcopenia and frailty formed the dominant clinical focus. These spanned endocrine and biomarker correlates [59], mortality, frailty and physical-performance outcomes [137,140], cognition and incident dementia [139], heart failure [141], and validation of BIA against DXA [142]. Other topics included body composition in pre-frail older adults [143], PhA as a muscle-quality index [146], gut–microbiome associations [148], cancer-related sarcopenia and malnutrition [149,155], exercise and multidomain interventions [150,153], and sarcopenic obesity [154]. Obesity and adiposity constituted the second cluster, ranging from ketogenic dietary intervention [135] and school-based pediatric programs [144] to anthropometric and adiposity indices [138,152]. These indices were associated with cognition [60], cardiovascular disease [157], and heart failure [158]. The remaining clinical areas included athletic populations [89,136] and hepatic disease [147]. Two additional articles focused primarily on research infrastructure and methodology: automated CT segmentation [145], wearable impedance biosensors [156], and the profile of a large population cohort [151].

The role assigned to BIA as a body-composition measurement technique was more central here than in the review literature: it was the primary measurement technique in two thirds of the studies and of marginal relevance in only two. In those two, adiposity was assessed by DXA and BIA figures were quoted only for comparison with results from other authors [152], or the impedance measurement was confined to the electrode–skin interface of a novel wearable biosensor rather than to body composition [156]. The contrast is informative: whereas high-impact reviews and guidelines tend to list BIA as one option among several accepted reference methods, the original research reaching comparable citation levels is largely built on BIA as the measurement instrument of record. This asymmetry in the role assigned to BIA between the two groups was confirmed by contingency-table analysis. Table 5 shows the cross-tabulation together with the associated χ^2^ statistics.

An article was therefore roughly five times more likely to treat BIA as the primary measurement technique if it reported primary data than if it was a review or guideline document.

The same instrument-level reading of Table A2 reveals an asymmetry with the review literature. PhA is used in only five of the 27 original studies [89,143,146,150,156], and in one of them the impedance measurement concerns the electrode–skin interface of a wearable sensor rather than the body [156]; equation-derived muscle-mass indices underpin fourteen studies.

The most-cited primary-data studies continue to rely predominantly on model-dependent body-composition estimates, despite the growing use of raw bioelectrical descriptors in a smaller subset of studies. The transition toward raw descriptors is therefore more evident in reviews and consensus documents than in the current body of highly cited primary research.

Device provenance is also highly concentrated: the analyzers used come from only two manufacturers, eight of them InBody platforms [59,137,142,143,148,149,151,154] and four Tanita [140,147,155,157]. Because both rely on proprietary, device-specific equations, a substantial share of the high-impact evidence is anchored to two commercial calibrations.

Scale and provenance are equally uneven. Sample sizes are traceable for 23 of the 27 studies and span four orders of magnitude, from 50 participants in an exercise intervention [150] to 468,333 in a UK Biobank analysis [157], with a median of 1417. Three population-based resources contribute about 96% of the pooled participants: two analyses of the same UK Biobank cohort [147,157] and one Norwegian population survey [151]. Most of the statistical weight of this group therefore comes from secondary analyses of pre-existing cohorts rather than from purpose-designed BIA studies. Where the study population can be located, seven were conducted in East Asian populations [59,137,138,142,146,148,154], against two in the United Kingdom [147,157], one in Norway [151] and one in Ireland [153]. The sarcopenia evidence therefore derives largely from populations in which the Asian Working Group for Sarcopenia (AWGS) criteria and cutoffs apply, whereas the large-cohort adiposity evidence is European. This constrains the transferability of population- and device-specific thresholds between the two clusters. Hard endpoints also remain scarce: mortality or survival is examined in four studies [137,141,149,155] and incident disease in three [139,147,157], with the remainder reporting cross-sectional or short-term associations.

### 5.4. Shared Findings and Evidence Gaps

The chronological distribution peaked in 2021 (nine studies), followed by 2022 (six), 2023 (five), 2020 (four), 2024 (two), and 2025 (one), with the decline in the final years again reflecting the citations-per-year threshold.

Table 6 regroups the 64 articles into seven broad clinical areas by group.

The obesity/adiposity literature was disproportionately drawn from large-cohort primary-data studies rather than reviews (Fisher exact OR = 6.12, *p* = 0.029), consistent with its dependence on population-scale resources such as UK Biobank. The methodology and PhA literature, by contrast, was almost exclusively contained in the review literature.

Taken together, these findings suggest that the recent high-impact BIA literature follows a characteristic translational trajectory. Methodological development is largely driven by review articles and consensus documents, whereas primary investigations focus on validating the clinical usefulness of already established body-composition metrics within a limited number of disease areas, particularly sarcopenia and obesity.

The adoption of raw bioelectrical biomarkers in clinical research remains comparatively limited, despite their growing prominence in methodological reviews. At the same time, several settings in which reliable body-composition assessment is most needed, notably critical illness, advanced renal disease and acute care, remain substantially underrepresented. These patterns indicate that future research priorities should extend beyond incremental validation studies toward broader clinical implementation and the standardization of raw bioelectrical parameters across diverse patient populations. These barriers are not confined to instrumentation. The standardization of electrode placement and of pre-measurement conditions, population-specific reference values, transparent analytical pipelines and clinically interpretable thresholds are equally necessary before raw bioelectrical parameters can be used in routine practice.

### 5.5. Are the Identified Gaps Being Addressed?

The bibliometric analysis presented in Section 5.1, Section 5.2, Section 5.3 and Section 5.4 selects for citation impact and therefore cannot describe the most recent literature, in which citations have not yet had time to accumulate. To establish whether the gaps identified above are being addressed, a complementary search was performed on 30 July 2026 using the same technique and nutrition equations, restricted to the Article document type and to the 2025–2026 publication window, and without any citation threshold. The search retrieved 1672 records, of which 14 were discarded as non-human studies belonging to animal or food science and three because their print year fell outside the window, leaving 1655 articles.

Each record was assigned to a single clinical area from its full text, using the categories of Table 6 with two refinements. First, the residual other clinical category was resolved into critical/acute care and renal disease, with the two settings identified as underrepresented in Section 5. Second, records spanning more than one area were assigned by a fixed priority order: critical/acute care, renal disease, oncology, malnutrition, sports, sarcopenia/frailty, obesity/adiposity, methodology, and other. The same rule was applied retrospectively to the 64 articles of Section 5, so that the two distributions are directly comparable. Table 7 reports the resulting distributions side by side. Both distributions are expressed as shares of their own corpus. They therefore describe how bioimpedance research is distributed across clinical areas rather than how intensively the technique is used within each area: an area that is large in the nutrition literature as a whole will tend to contribute a large share irrespective of its adoption of bioimpedance. Normalizing each area to its own total output would require field-level denominators outside the scope of the present search. The comparisons that follow are therefore drawn between the two corpora within a given area, and the shares are not read as a ranking of uptake between areas.

Critical and acute care remains the setting with the smallest share of the corpus: 22 of the 1655 recent articles (1.3%), essentially unchanged from the single article (1.6%), were retained in the most-cited corpus. This is the clearest outcome of the comparison, and it is robust to the differences between the two datasets, since the proportion is equally marginal under either selection criterion. Renal disease follows the opposite trajectory, rising from two articles (3.1%) to 138 (8.3%), which is the largest proportional increase in any area. The two settings that Section 5.1, Section 5.2, Section 5.3 and Section 5.4 identified jointly as underrepresented have therefore diverged: the share of the corpus occupied by renal disease is expanding, whereas that of intensive and acute care is not.

Within the areas that were already well-covered, obesity and adiposity has become the largest single category of the recent corpus (35.4%), ahead of sarcopenia and frailty (24.4%), reversing the order observed in the most-cited corpus. Because the two areas differ in the overall volume of nutrition literature they generate, this ordering locates where recent bioimpedance articles are concentrated and does not by itself indicate a higher uptake of the technique in obesity than in sarcopenia. The contraction of the methodological category, from 17.2% to 6.0%, should not be read as a decline in methodological activity. The corpus of Section 5.1, Section 5.2, Section 5.3 and Section 5.4 was selected by citations per year without restriction of document type and therefore concentrates reviews, guidelines and consensus documents, which is precisely where methodological work is published, whereas the present corpus contains original articles only. The comparison is accordingly informative for clinical areas, where both corpora indicate where research effort is directed, and uninformative for categories whose size depends on document type.

Raw bioelectrical parameters are explicitly reported in 306 of the 1655 recent articles (18.5%). Their use concentrates precisely where fluid status is least stable: 45% of the critical and acute care articles and 34% of the malnutrition articles report PhA or vector analysis, against 4% of the obesity articles. Unlike the column shares of Table 7, these are proportions computed within each area and are therefore independent of the relative size of the areas. The direction is unambiguous: the adoption of raw bioelectrical descriptors, described as comparatively limited in the most-cited literature, is progressing, and it is progressing fastest in the settings where fluid redistribution most rapidly invalidates regression-based estimates.

## 6. Concluding Remarks

The scope of BIA has widened considerably since its early use as an estimator of TBW. Its value nevertheless rests on an indirect measurement: what is recorded is the electrical response of biological tissues, which is then converted into physiological variables through predictive models. The accuracy of the resulting compartment estimates therefore depends as much on the reference method used for calibration and on the population from which the equation was derived as on the precision of the electrical measurement itself.

The cumulative publication record places the maturity of BIA technologies in perspective. Weighted logistic models reproduce the growth of five of the most widely used body composition techniques with coefficients of determination at or above 0.999. The weighted logistic analyses should be interpreted as descriptive, model-dependent analyses rather than as established forecasts of future publication activity. Very high R^2^ values are not in themselves evidence of predictive validity when fitting smooth cumulative publication counts. However, a logistic curve accumulates half of its asymptote at the inflection point, which falls beyond the observation window for BIA. Under the fitted model, it is likely that fewer than half of the BIA articles it projects have so far been published.

Interpreting a bioimpedance measurement requires attention to both tissue electrical properties and instrumental limits. EP, skin impedance, parasitic capacitances, distributed electromagnetic effects, and frequency-dependent dielectric phenomena all influence the measured impedance and bound the physiological information that can be extracted. This is why extending the measurement toward both lower and higher frequencies remains technically challenging.

A second constraint lies in the equations rather than in the hardware. Anthropometric variables frequently contribute as much as, or more than, the electrical measurement, and MIF analysis makes that contribution explicit. This explains why instruments that record nearly identical impedances can return different body composition estimates. The consequence is not uniform across patients. FM is obtained by subtracting FFM from body weight, so the error of the FFM estimate passes to FM in full. Both the constant-hydration assumption and the cohorts used for calibration are least representative at high BMI, so accuracy is lowest at the upper end of the BMI range. We can conclude that greater transparency regarding proprietary prediction equations would facilitate independent validation and improve inter-device comparability. It would also clarify the standing of outputs such as VAT and BMC, which commercial devices report routinely and clinical studies already use as endpoints, but for which no quantitative validation was identified in this review.

Considerable progress has also been achieved in the direct interpretation of raw bioelectrical variables, particularly PhA and BIVA. These parameters provide clinically relevant information without relying on regression equations and are therefore not subject to the calibration bias that grows with adiposity. Together with advances in multifrequency measurements and segmental analysis, these developments are progressively shifting the field from purely empirical body composition estimation toward a more mechanistic understanding of tissue physiology.

Beyond body composition assessment, the electrode technologies, BWs and bioimpedance imaging mark the most likely direction of expansion. The regional distribution of change carries clinical information of its own: muscle wasting is uneven across limbs. Trunk and fluid overload accumulates in dependent regions. The weight that conventional imaging has acquired in nutrition research is evidence of that interest. Bioimpedance imaging brings regional resolution within reach of a radiation-free measurement that can be repeated at the bedside. It is therefore of direct use to sarcopenia and cachexia, to fluid management in dialysis and critical care, to refeeding in malnutrition, and to neuromuscular rehabilitation. Its present limit is spatial resolution. Closing that gap would be consequential. DXA needs fixed, high-cost instrumentation, radiation safety procedures and trained operators. Its dose is low, but it still limits how often a measurement can be repeated, particularly in children, in pregnancy and in longitudinal follow-up. A bioimpedance measurement takes a fraction of the time. It can be made at the bedside or at home, and it can be repeated daily.

Bioimpedance may offer limited accuracy for some body-composition parameters, yet it provides major advantages for research. With BIA, sample size is no longer the primary constraint: large population cohorts already incorporate bioimpedance measurements, and individual studies can include hundreds of thousands of participants, a scale that is difficult to achieve with conventional imaging techniques. Prediction equations may introduce systematic errors. But these errors are largely canceled when they remain stable within individuals and the outcome of interest is change rather than absolute value. Segmental PhA and BIVA are already used this way in dialysis, critical care, nutrition, rehabilitation, and sport.

The most-cited literature of the recent years converges on sarcopenia and, secondarily, on obesity and adiposity, and rests predominantly on narrative and consensus documents rather than on pooled quantitative synthesis. Critical illness and renal disease, where fluid redistribution is fastest and least predictable, occupy the smallest share of that corpus. The primary evidence is also concentrated on the analyzers of only two manufacturers, both relying on proprietary equations, so a large part of it rests on two commercial calibrations. The literature published in 2025 and 2026 shows that this picture is already changing unevenly. Renal disease has risen from 3% to 8% of the bioimpedance corpus, and obesity has displaced sarcopenia as the largest single area of application within it, so the technique is now applied most intensively where the equations are least accurate. Raw bioelectrical parameters now appear in nearly one fifth of recent articles, and most often in critical care and malnutrition, that is, precisely where fluid status is least stable. Critical and acute care, however, remains marginal.

Two limitations qualify these conclusions. First, the statements concerning measurement principles, the dependence of compartment estimates on prediction equations, and the clinical use of raw bioelectrical parameters derive from the published evidence reviewed here. The statements concerning the maturity, distribution and expected trajectory of each technique derive instead from the bibliometric analysis. They describe research activity only and should not be read as evidence of clinical validity. Second, the bibliometric results depend on the database, the document type and the search terminology used. Web of Science indexes journals selectively and its coverage of early work is less complete, so publication counts reflect indexed visibility rather than historical priority. Applications described with heterogeneous terminology may also be underrepresented. Reproducing the analysis in other databases, or with different search equations, would be expected to shift the absolute counts. It is unlikely, however, to alter the relative ordering of the five techniques.

## Figures and Tables

**Table 1:** Search strategy in Web of Science detailing the Topic Search (TS) equations for each body composition technique and the nutrition domain. Each search combined the technique equation listed below with a common nutrition equation: TS = (nutrition* OR malnutrition OR malnourish* OR undernutrition OR undernourish* OR obes* OR overweight OR sarcopeni* OR cachex* OR adipos*). The total formula is (technique equation) AND (nutrition equation) AND DT = (Article).

Technique	Search Term for Technique (TS=)
BIA	TS = (“bioimpedance*” OR “bioimpedance*” OR “bioelectric* impedance*” OR “impedance vector analysis” OR BIA OR BIVA OR (“phase angle” AND (“bioimpedance*” OR “bioimpedance*” OR “bioelectric* impedance*”)))
DXA	TS = (DXA OR DEXA OR “dual-energy X-ray absorptiometr*” OR “dual-energy X-ray absorptiometr*”)
CT	TS = (“computed tomograph*” OR “computerized tomograph*” OR “computerised tomograph*” OR “CT scan*” OR “CT-derived” OR “CT image*”)
MRI	TS = (MRI OR “magnetic resonance imag*” OR “MR imaging” OR “magnetic resonance tomograph*”)
US	TS = (ultrasound* OR ultrasonograph* OR sonograph* OR echograph*) NOT TS = (“ultrasound-assisted” OR ultrasonicat* OR sonicat* OR “ultrasonic extraction”)

**Table 2:** Three examples of bioimpedance prediction equations. MIF: measurement-independent factor; *H*: body height in cm; *R*: electrical resistance in Ohm at 50 kHz; *Xc*: absolute value of reactance in Ohm at 50 kHz; *Wt*: body weight in kg; *BMI*: body mass index; *Sex* factor: 1 for males, 0 for females. *R*^2^: coefficient of determination. *SEE*: standard error of estimation.

Author	Equation	MIF	Participants	Reference	*R* ^2^	*SEE*
Lukaski et al. [38]	TBW (l) = 0.377·*H*^2^/*R* + MIF	0.140·*Wt* − 0.080·*Age* + 2.90·*Sex* + 4.65	*N* = 59 females and *N* = 51 males, 20–73 years	Isotope dilution	0.975	1.50 l
Kyle et al. [39]	ALSM (kg) = 0.267·*H*^2^/*R* + 0.058·*Xc* + MIF	0.095·*Wt* − 0.012·*Age* + 1.909·*Sex* − 4.211	Healthy men (*N* = 246, *BMI* 25.3 kg/m^2^) and women (*N* = 198, *BMI* 24.1 kg/m^2^), ages: 20–94 years	DXA	0.98	1.12 kg
Hughes et al. [40]	FFM (kg) = 0.432·*H*^2^/*R* + MIF	0.269·*Wt* − 0.086·*Age* − 6.422·(1 − *Sex*) + 16.429	147 Indigenous Australian adults with a median BMI of 31 kg/m^2^	DXA	0.94	3.33 kg

**Table 3:** Commercially available analyzers. DSM: Direct Segmental Multifrequency; MF: Multifrequency; SF: Single frequency.

Manufacturer	Model	Year	Electrodes	Type	Frequencies (kHz)
Akern	BIA 101 Anniversary	2020	4	SF	50
Data Input	Nutriguard-MS	2010	4	MF	5, 50, 100
Bodystat	QuadScan 4000	2021	4	MF	5, 50, 100, 200
InBody	InBody 770	2015	8	DSM	1, 5, 50, 250, 500, 1000
InBody	InBody 270	2016	8	MF	20, 100
InBody	InBody 970S	2025	8	DSM	5, 50, 250, 500, 1000, 3000
InBody	BWA 2.0S	2025	8	DSM	1, 5, 50, 250, 500, 1000, 3000
InBody	S10	2013	8	DSM	1, 5, 50, 250, 500, 1000
Seca	mBCA 515	2013	8	MF	19 freqs. between 1 and 1000
Seca	mBCA 555	2020	8	MF	19 freqs. between 1 and 1000
Tanita	MC-980MA Plus	2014	8	MF	1, 5, 50, 250, 500, 1000
Tanita	MC-780MA	2014	8	MF	1, 5, 50, 250, 500, 1000
Tanita	BC 601	2010	8	SF	50

**Table 4:** Citations per year (cites/year), by group and by the role assigned to BIA. SD: standard deviation. ^1^ The two articles assigning BIA a marginal role were excluded from the role comparison given their small number.

Grouping	n	Median	Mean	SD
Review-type articles	37	35.23	77.80	164.10
Primary-data studies	27	24.46	31.29	16.44
BIA role: primary ^1^	28	26.24	31.00	12.86
BIA role: complementary ^1^	34	34.67	81.87	171.00

**Table 5:** Contingency table of the role assigned to BIA (primary vs. complementary/marginal) by group. Fisher’s exact test on the same table gave an odds ratio of 0.19 (*p* = 0.002).

Group	Primary, n (%)	Complementary/Marginal, n (%)	χ^2^ (*df* = 1)	*p*-Value
Review-type (n = 37)	10 (27.0%)	27 (73.0%)	8.42	0.004
Non-review (n = 27)	18 (66.7%)	9 (33.3%)	–	–

**Table 6:** Distribution of articles across broad clinical areas, by group.

Clinical Area	Non-Review, n	Review-Type, n
Sarcopenia/frailty	14	12
Obesity/adiposity	7	2
Methodology (incl. PhA)	1	11
Sports/athletes	2	2
Malnutrition	0	3
Oncology	0	3
Other clinical	3	4

**Table 7:** Distribution of articles across clinical areas in the most-cited corpus of Section 5.1, Section 5.2, Section 5.3 and Section 5.4 and in the 2025–2026 literature. Areas were assigned from the full text using a fixed priority order; percentages are column shares.

Clinical Area	Most-Cited Corpus, 2020–2026 (n = 64)	Recent Literature, 2025–2026 (n = 1655)
Sarcopenia/frailty	22 (34.4%)	403 (24.4%)
Obesity/adiposity	9 (14.1%)	586 (35.4%)
Methodology (incl. PhA)	11 (17.2%)	100 (6.0%)
Sports/athletes	7 (10.9%)	107 (6.5%)
Oncology	5 (7.8%)	143 (8.6%)
Malnutrition	3 (4.7%)	119 (7.2%)
Renal disease	2 (3.1%)	138 (8.3%)
Critical/acute care	1 (1.6%)	22 (1.3%)
Other clinical	4 (6.3%)	37 (2.2%)
**Total**	**64 (100%)**	**1655 (100%)**

## Data Availability

The data required to reproduce the tables presented in this paper are available from J.M.M. upon reasonable request. The data analyzed in this study are publicly available from the National Health and Nutrition Examination Survey (NHANES) 1999–2000, conducted by the National Center for Health Statistics (NCHS), Centers for Disease Control and Prevention (CDC).

## Supplementary Materials

The following supporting information can be downloaded at https://www.mdpi.com/article/10.3390/nu18172889/s1, Supplementary_Files.zip, containing Raw Web of Science literature search exports (5625 records), as retrieved directly from the Web of Science search interface, source data spreadsheets and MATLAB (v. 2025b version) plotting scripts for manuscript figures.

## Abbreviations

4C	Four-compartment model
ALSM	Appendicular lean soft mass
ALST	Appendicular lean soft tissue
ASMM	Appendicular skeletal muscle mass
ASPEN	American Society for Parenteral and Enteral Nutrition
AWGS	Asian Working Group for Sarcopenia
BCM	Body cell mass
BIA	Bioelectrical impedance analysis
BIS	Bioimpedance spectroscopy
BIVA	Bioelectrical impedance vector analysis
BMC	Bone mineral content
BMI	Body mass index
BMR	Basal metabolic rate
BPE	BIA prediction equation
BW	Bioimpedance wearable
CT	Computed tomography
DSM	Direct segmental multifrequency
DXA	Dual-energy X-ray absorptiometry
ECW	Extracellular water
EIM	Electrical impedance myography
EIT	Electrical impedance tomography
EP	Electrode polarization
ESPEN	European Society for Clinical Nutrition and Metabolism
FFM	Fat-free mass
FFMI	Fat-free mass index
FM	Fat mass
FMI	Fat mass index
GLIM	Global Leadership Initiative on Malnutrition
ICW	Intracellular water
MF-BIA	Multifrequency bioelectrical impedance analysis
MIF	Measurement-independent factor
MRI	Magnetic resonance imaging
PEDOT:PSS	Poly(3,4-ethylenedioxythiophene):poly(styrene sulfonate)
PhA	Phase angle
SBIA	Segmental bioimpedance analysis
sBIVA	Specific bioelectrical impedance vector analysis
SEE	Standard error of estimation
SMM	Skeletal muscle mass
TBW	Total body water
US	Ultrasound
VAT	Visceral adipose tissue
WEIT	Wearable electrical impedance tomography

## Appendix A

**Table A1:** Review articles, systematic reviews/meta-analyses, and consensus guidelines on BIA in clinical nutrition among most-cited publications (>20 cites/year) in the field, ordered by year of publication, from 2020 to 2026.

Authors	Study Type/Area	BIA Usage	Summary of Main Findings
Chen, L.-K., et al. (2020) [103]	Consensus statement/diagnostic guidance—Sarcopenia (diagnosis/consensus)	Complementary—BIA endorsed alongside DXA for the muscle-mass criterion, with height-adjusted cutoffs of <7.0 kg/m^2^ (men) and <5.7 kg/m^2^ (women).	AWGS 2019 consensus update: retains the definition of sarcopenia but revises the algorithm and cutoffs (handgrip <28 kg men/<18 kg women; gait <1.0 m/s), adds separate community and hospital pathways with upfront screening, and introduces ‘possible sarcopenia’ for primary care, with exercise plus nutrition as treatment mainstays.
Ponti, F., et al. (2020) [61]	Narrative review—Body composition methodology (imaging/aging)	Complementary—BIA cited only as a low-cost but imprecise alternative to imaging; the review centers on DXA, CT, MRI and ultrasound.	Review of body-composition change with aging: total and visceral fat rise while lean and bone mass fall, increasing the risk of cardiovascular disease, diabetes, disability and sarcopenia. Imaging is far more accurate than BMI or anthropometry, but cost, availability and radiation limit routine clinical use.
Walowski, C. O., et al. (2020) [104]	Narrative review—Body composition methodology (muscle mass)	Complementary—BIA compared with DXA and CT as a source of ASMM cutoffs.	Review of 64 studies compiling SMM reference values for sarcopenia diagnosis, showing wide variation in cutoffs across methods. Results are comparable only with the same method, device and reference population, normalization for FM is needed in obesity, and a unified nomenclature is essential. BIA validity was device- and population-dependent: acceptable for whole-body SMM but poor for limb compartments, and the widely used Janssen equation is not validated in Asian populations.
Mattiello, R., et al. (2020) [88]	Systematic review and meta-analysis (PRISMA; PROSPERO-registered)—Body composition methodology (PhA)	Primary—pooled PhA reference values from 50 kHz BIA in all studies.	Meta-analysis of 46 studies and 249,844 healthy subjects (80% female) pooling PhA by sex and 13 age groups. PhA rises during growth from 3.6° at 0–2 y to a peak of 7.3° at 16–18 y in males and from 3.7 to 6.4° in females, plateaus through adulthood (to ~38 y in males and ~48 y in females) and declines in old age (5.3 and 5.4° at >80 y); males exceed females in every group except 0–2 y and >80 y. Heterogeneity was very high (*I*^2^ 97–100%), so standardized BIA reporting is needed. Proposed clinically meaningful differences: 1.0° (males) and 0.90° (females).
Sheean, P., et al. (2020) [54]	Clinical practice guideline (based on systematic review)—Body composition methodology (validity in clinical populations)	Complementary—23 BIA studies reviewed, including PhA derived from raw R and Xc.	ASPEN clinical practice guideline based on a systematic review (7375 studies screened) of body-composition methods in adult clinical populations. DXA receives a strong recommendation for FM assessment, its validity for lean mass remains undetermined, and no recommendation was possible for ultrasound. For BIA, despite good correlations with DXA, CT and MRI, no recommendation on validity could be issued because proprietary manufacturer-specific equations prevented data pooling. PhA, obtained directly from raw R and Xc, is highlighted as an emerging parameter still requiring validation against CT-derived skeletal muscle.
Perez–Campos Mayoral, L., et al. (2020) [105]	Narrative review—Obesity (phenotypes/biomarkers)	Complementary—BIA as one of several body-composition tools in sarcopenia assessment.	Review of adult obesity phenotypes (metabolically healthy, metabolically abnormal, metabolically obese normal-weight and sarcopenic obese) and their inflammatory, adipokine and genetic markers. No consensus exists on terminology or diagnostic criteria and current biomarkers have low predictive value. BIA use is limited by lack of standardization, inappropriate FFM equations and the need for population-specific cutoffs.
Ikizler, T. A., et al. (2020) [106]	Clinical practice guideline—Chronic kidney disease (nutrition)	Complementary—MF-BIA suggested for body composition in maintenance hemodialysis, measured at least 30 min after dialysis (2C); insufficient evidence to recommend BIA in non-dialysis CKD and peritoneal dialysis (2D).	KDOQI/Academy of Nutrition and Dietetics 2020 GRADE-based nutrition guideline for CKD stages 1–5, dialysis and transplant recipients, covering nutritional assessment, medical nutrition therapy, protein and energy intake, supplementation, micronutrients and electrolytes. In maintenance hemodialysis it suggests bioimpedance, preferably MF-BIA, performed at least 30 min after dialysis to allow fluid redistribution (2C); MF-BIA agreed well with DXA for FM and FFM and predicted hospitalization and survival. In non-dialysis CKD and peritoneal dialysis, the evidence was judged insufficient to recommend BIA (2D), hydration widening the disagreement with DXA. Higher PhA reflected better nutritional status and predicted long-term survival, whereas BIVA related to nutritional intake markers only in well-nourished patients.
Holmes, C. J., and Racette, S. B. (2021) [107]	Narrative review/overview—Body composition methodology (general)	Complementary—BIA, segmental BIA and PhA among the available body-composition methods.	Review of body-composition assessment in obesity, sarcopenia, osteoporosis, malnutrition, cancer cachexia and lymphedema. DXA is the current preferred criterion method, no single gold standard exists, and combining methods is recommended, particularly for tracking longitudinal change. BIA accuracy depends on device-specific equations and hydration, diverges from DXA at higher BMI, and BIA is contraindicated with implantable electronic devices; PhA indicates cellular integrity, water distribution and nutritional status, and segmental BIA is useful for monitoring lymphedema.
Campa, F., et al. (2021) [108]	Narrative review—Sports/athletes	Primary—review centered on BIA and BIVA in athletes.	Review of BIA in athletes. Foot-to-hand technology at 50 kHz with athlete- and sport-specific equations is the reference approach and predicts TBW, ECW, FFM and segmental lean soft-tissue well, whereas general-population formulas are unreliable. PhA is a qualitative marker of cellular integrity, positively associated with the ICW/ECW ratio, and is recommended for within-athlete longitudinal monitoring rather than between-athlete comparison. BIVA, validated against dilution techniques and DXA, classifies hydration through sport-specific tolerance ellipses and discriminates elite from sub-elite athletes.
Di Vincenzo, O., et al. (2021) [109]	Systematic review (PRISMA)—Sarcopenia (PhA)	Primary—systematic review of BIA-derived PhA in sarcopenia.	Review of 13 studies and 7668 subjects (community-dwelling and hospitalized older adults, cancer, COPD, cirrhosis, kidney transplant). PhA and sarcopenia independently predicted survival, and low PhA predicted incident falls and disability whereas individual sarcopenia components did not. Seven of eight studies found lower PhA in sarcopenia (−4.9% to −25.7%) and low PhA up to doubled sarcopenia prevalence. ROC cutoffs ranged 3.55–5.05° in the full text (4.05–5.05° as reported in the abstract), with sensitivity ~70% and specificity 52–70%, judged insufficiently reliable; PhA correlated weakly to moderately with SMM, grip strength and gait speed.
Kasper, A. M., et al. (2021) [110]	Narrative review—Sports/athletes (methodology)	Complementary—BIA and BIS, with BIS preferred for applying Cole modeling rather than simple regression.	Review of body-composition methods in applied sport practice. DXA is preferred for bone mineral content and regional fat and lean estimates but is logistically demanding, whereas skinfolds are least affected by day-to-day variability; standardized methods can track longitudinal change. BIA and BIS are valued for speed, portability and cost, with BIS preferred over BIA because it relies on Cole modeling and mixture theory rather than simple regression equations. Both are limited by hydration, temperature, electrode placement and population-specific assumptions, by scarce and conflicting athlete validation against DXA, and by large inter-device variability.
Moonen, H. P. F. X., and Van Zanten, A. R. H. (2021) [111]	Narrative review (‘Current Opinion’ article)—Critical illness (body composition)	Primary—review dedicated to BIA, PhA and BIVA in critically ill patients.	Review of BIA in critically ill ICU patients. PhA is consistently low; admission PhA predicts 28-day, 90-day and 12-month mortality, ICU stay and ventilation time (<5.58° = high nutrition risk). BIVA predicts mortality better than cumulative fluid balance; ECW/TBW >0.40 indicates overhydration and 0.413 predicts 28-day mortality on CRRT. Raw R, Xc and PhA stay interpretable, whereas equation-derived FFM and BCM depend on water-distribution assumptions. Not validated in critical illness: no ICU cutoffs or reference values.
Lukaski, H., and Raymond-Pope, C. J. (2021) [112]	Narrative review—Sports/athletes	Complementary—BIA discussed alongside DXA for measuring total and regional fat and lean mass, with emphasis on minimizing measurement error and standardizing protocols.	Review of functional body composition in adult athletes, linking purposeful fat and lean-mass measurement to muscle function, sport-specific performance, injury risk and return-to-sport decisions, and calling for research beyond the prevailing descriptive studies.
Aprile, G., et al. (2021) [113]	Narrative review—Oncology (nutrition)	Complementary—BIA as a second-level body-composition tool alongside CT/MRI-derived SMI.	Review on nutritional care before and during cancer treatment: malnutrition affects ~50% of patients yet many are never assessed, and nutritional status may influence the efficacy of chemotherapy, targeted agents and immune checkpoint inhibitors. Early, proactive screening throughout the care pathway is essential. BIA is described as novel and widely used, although image-based methods appear more accurate.
Bossi, P., et al. (2021) [114]	Narrative review—Oncology/cachexia–sarcopenia	Complementary—BIA, BIVA and PhA listed among the tools available for assessing hydration, cell mass and outcome prediction within nutritional assessment.	Review of malnutrition, cachexia and sarcopenia in cancer: malnutrition affects up to 75% of patients and pre-therapeutic sarcopenia 38.6% (52.8% in lung cancer), both worsening with advanced stage and with locoregional and systemic treatment. Recommends early screening, periodic reassessment and proactive nutritional support. BIA-derived tools, including BIVA and PhA, are listed among the methods available for assessing hydration and cell mass and for predicting outcomes.
Petermann–Rocha, F., et al. (2022) [115]	Systematic review and meta-analysis (PROSPERO-registered)—Sarcopenia (epidemiology)	Complementary—BIA was the most frequently used muscle-mass instrument across the included studies, ahead of DXA and CT.	Review of 151 studies (n = 692,056) estimating global sarcopenia prevalence under six diagnostic classifications: pooled estimates ranged from 10% (EWGSOP2) to 27% (muscle mass alone) and severe sarcopenia from 2% to 9%, showing that the lack of a consensual criterion hinders comparability. BIA was the most frequently used instrument to assess muscle mass (137 studies), ahead of DXA (121) and CT (11).
Barazzoni, R., et al. (2022) [116]	Consensus/expert guidance—Malnutrition (diagnosis/muscle mass)	Complementary—SF-BIA, MF-BIA and segmental devices, plus PhA from raw R and Xc, recommended for the muscle-mass criterion alongside DXA and CT.	GLIM consortium consensus with five expert-opinion statements on assessing the muscle-mass criterion for malnutrition diagnosis, endorsing validated ethnic- and sex-specific cutoffs and, where technology-based methods are unavailable, anthropometry and physical examination. Cutoffs separating moderate from severe reduction are flagged as an urgent research priority. BIA is low cost, portable, repeatable and predictive of clinical outcomes, but limited by population- and device-specific equations, overestimation under overhydration and the need to validate single-frequency, multifrequency and segmental devices separately.
Shu, X., et al. (2022) [117]	Systematic review and meta-analysis—Sarcopenia (dialysis/CKD)	Complementary—muscle mass assessed by BIA or BIS in a minority of the included studies.	Review of 30 studies and 6162 dialysis patients: pooled sarcopenia prevalence 28.5%, with no significant differences by diagnostic criteria, dialysis modality, age or dialysis duration, and consistently higher mortality (HR 1.61–2.04). Standardized diagnostic criteria are needed. Muscle mass was assessed by DXA (17 studies), BIA (8) and BIS (5), with a wide range of cutoffs used to define low muscle mass.
Ackermans, L. L. G. C., et al. (2022) [118]	Narrative review—Sarcopenia (assessment methodology)	Complementary—BIA, PhA, the ECM/BCM index, BIVA, BIS and EIM reviewed, with CT as the gold standard.	Review mapping sarcopenia assessment tools to three clinical scenarios—screening, diagnosis and longitudinal monitoring—and rating usability by sensitivity and specificity. For screening it recommends SARC-F with calf or mid-arm circumference plus grip strength or gait speed, with CT as the diagnostic gold standard. BIA is non-invasive, affordable and valid for single assessments and longitudinal monitoring; PhA is a reliable prognostic marker of malnutrition and the ECM/BCM index an early predictor, whereas BIVA, BIS and EIM are insufficiently validated for routine clinical use.
Campa, F., et al. (2022) [119]	Systematic review (PRISMA-guided)—Sports/athletes (methodology)	Primary—systematic review of BIA and BIVA against reference methods (DXA, underwater weighing, dilution techniques, 4C model, ADP, CT).	Review of 42 studies and 2978 athletes. BIA was inaccurate for %FM with every technology, whereas foot-to-hand with athlete-specific equations agreed well for FFM, TBW, ECW and ICW; generalized equations and direct segmental devices underestimated FFM and body fluids. Classic BIVA vector length correlated negatively with TBW and specific BIVA positively with %FM, while PhA correlated positively with the ICW/ECW ratio, supporting it as an indicator of cellular health and fluid distribution. Valid only with foot-to-hand technology, athlete-specific equations and sport-specific tolerance ellipses.
Prado, C. M., et al. (2022) [120]	Narrative review—Muscle health/malnutrition	Complementary—BIA for muscle-mass estimation, with PhA as a prognostic marker.	Review from the 119th Abbott Nutrition Research Conference on muscle health and malnutrition: pathophysiology, populations at risk, assessment of muscle mass and function, and interventions, advocating multimodal care that combines protein, leucine, HMB, vitamin D and *n*-3 PUFA with resistance exercise. Combined resistance exercise, protein and vitamin D improved PhA in older adults. BIA estimates muscle mass through population-, equation- and device-specific formulas, carrying a risk of error; PhA reflects cell-membrane integrity linked to inflammation, oxidative stress and survival, with low values associated with higher sarcopenia prevalence, while cutoffs are population- and device-specific and values are influenced by fluid balance, obesity and edema.
Seron–Arbeloa, C., et al. (2022) [121]	Narrative review—Malnutrition (general)	Complementary—BIA for TBW, body cell mass and FM, with PhA as the most widely used bioimpedance parameter.	Review of nutritional screening and assessment tools (NRS-2002, MUST, MNA-SF, NUTRIC, SGA, ASPEN/AND, ESPEN, GLIM) across hospitalized, elderly, critically ill, cancer and CKD populations. No gold standard exists: screening and assessment must be combined to enable early intervention and reduce complications and costs. BIA is simple, inexpensive and non-invasive but unsuitable in fluid overload and sensitive to hydration and interobserver variability; low PhA values are linked to frailty, malnutrition, disability and mortality.
Bellido, D., et al. (2023) [93]	Narrative review with bibliometric analysis—Body composition methodology (PhA)	Primary—review centered on PhA and raw bioelectrical parameters.	Review of PhA research (>350 publications since 2004, >50% European). Raw parameters (R, Xc, PhA) avoid regression-equation bias and act as prognostic biomarkers; SF-BIA at 50 kHz with tetrapolar placement predominates, and BIVA assesses hydration and cell mass without predictive equations. Low PhA predicts mortality, complications, longer hospital stay and malnutrition; standardized PhA adjusts for age, sex, BMI, race and hydration. Limitations: inter-device variability and absence of universal cutoffs.
Branco, M. G., et al. (2023) [122]	Scoping review (PRISMA-ScR)—Oncology (body composition)	Primary—scoping review of BIA in oncology.	Review of 36 studies (~3000 patients under active cancer treatment). BIA is suitable, non-invasive and reproducible, though less accurate than DXA or CT. PhA is a prognostic indicator of membrane integrity and hydration: a 5.95° cutoff predicted 5-year survival in advanced head-and-neck cancer, and low PhA correlated with longer stay and worse survival in pancreatic, colorectal and lung cancer. BIVA assesses hydration and cell mass independently of body size, and FFM, FFMI, SMM and PhA add value in preoperative risk and complication prediction. Accuracy falls with edema, lymphedema, obesity and BMI extremes (≤16 or ≥35).
Lukaski, H. C., and Talluri, A. (2023) [92]	Narrative review—Body composition methodology (PhA)	Primary—review validating bioelectrical measurements, particularly PhA, as biomarkers of hydration and body cell mass.	Review validating raw bioelectrical measurements as biomarkers of body cell mass and hydration. With 50 kHz phase-sensitive devices, PhA = arctan(Xc/R) × 180/pi; R and Z track TBW while Xc indexes cells in fluid. Cell-membrane damage lowers Xc and PhA more than R; in surgical patients Xc explained more of the variance in fluid balance (R^2^ 81%) than R or Z (R^2^ 64%). PhA is positively associated with IGFBP-2 and negatively with NT-proBNP. Identical PhA values can differ by vector position, so BIVA must be read alongside cell mass.
Baek, J. Y., et al. (2023) [123]	Clinical practice guideline/expert consensus—Sarcopenia (diagnosis/guideline)	Complementary—direct segmental MF-BIA endorsed alongside DXA for ASMM, with PhA and the ECW/TBW ratio as adjuncts.	KWGS clinical practice guideline (two-phase Delphi, 40 panelists) for sarcopenia screening and diagnosis in older Korean adults, introducing a simplified diagnostic flow, prioritizing the SPPB and adding the concept of ‘functional sarcopenia’ (low strength plus low performance without documented muscle loss). Height-adjusted ASMM cutoffs are <7.0 kg/m^2^ (men) and <5.7 kg/m^2^ (women), and direct segmental MF-BIA correlates well with DXA.
Cappellari, G. G., et al. (2023) [124]	Consensus/expert proceedings—Sarcopenic obesity	Complementary—BIA as the alternative to DXA in SO, with PhA and BIVA as research avenues.	Proceedings and research-agenda document from the SOGLI expert panel (Rome, 2022) on sarcopenic obesity, setting out research priorities on definition, diagnostic criteria, epidemiology, pathophysiology and clinical outcomes. No primary data are presented. Research priorities for BIA in sarcopenic obesity: validating obesity-specific equations, correcting the over- or underestimation of FM and FFM caused by altered hydration, studying PhA as an equation-independent proxy for muscle mass and function, exploring BIVA in SO, and defining standardized population-specific cut-points.
Sweatt, K., et al. (2024) [125]	Narrative review—Obesity (BMI diagnosis)	Complementary—SF-BIA and MF-BIA for FM, muscle mass and body water, with MF-BIA more accurate for tracking change after weight loss.	Review on the limitations of BMI for diagnosing obesity: it should serve only as a screening test, with diagnosis combining anthropometry with clinical evaluation of complications under the ABCD framework. WHtR and WC/height^0.5 outperform BMI and waist circumference for predicting cardiometabolic risk, WHtR applying universal cutoffs across sex, race and muscle mass, while ABSI predicts all-cause mortality. BIA is non-invasive, quick and inexpensive, with MF-BIA more accurate than SF-BIA for tracking change after weight loss, but it underestimates FM in obesity, sarcopenia and metabolic syndrome and is affected by hydration; BIA-derived fat percentage shows a linear association with morbidity and mortality, unlike the U/J-shaped relationship seen with BMI.
Liu, D., et al. (2024) [126]	Narrative review—Sarcopenia (diagnosis and pathogenesis)	Complementary—BIA listed among the diagnostic tools under the EWGSOP2 and AWGS 2019 criteria.	Review of advances in sarcopenia: diagnostic criteria, signaling pathways (myostatin, AMPK, IGF-1/IIS, NF-kB), mitochondrial dysfunction, and exercise, nutritional and emerging pharmacological therapies, highlighting the absence of approved drugs and the need for individualized management.
Voulgaridou, G., et al. (2024) [127]	Critical/narrative review—Sarcopenia (diagnosis)	Complementary—BIA endorsed by EWGSOP2 and AWGS as an alternative to DXA, with PhA as a marker of muscle quantity and quality.	Review of sarcopenia diagnostic criteria and measurement techniques, comparing the definitions of the major working groups and the resulting variation in prevalence (~10% overall, up to 51% in nursing homes). Recommends measuring all three sarcopenia parameters with context-appropriate tools. BIA is cost-effective, portable and repeatable but estimates rather than measures muscle mass and may overestimate lean mass and underestimate FM; PhA lacks sarcopenia-specific cutoffs and its reference values vary by population, device and body position.
Pereira, J. P. D. C., et al. (2024) [128]	Systematic review and meta-analysis (PROSPERO-registered)—Body composition methodology (PhA/muscle quality)	Primary—meta-analysis of BIA-derived PhA as a muscle-quality marker.	Meta-analysis of 17 studies and 2710 participants (median age 67) on PhA as a marker of muscle quality; all studies at high risk of bias and certainty low to very low. PhA correlated moderately with CT muscle radiodensity and inversely with ultrasound echogenicity, weakly with the functional muscle-quality index. Accuracy for low radiodensity was good with cutoffs <5.10° (men) and <4.73° (women). PhA reflects myosteatosis rather than strength; device heterogeneity requires standardized PhA Z-scores.
Wilschanski, M., et al. (2024) [129]	Clinical practice guideline—Cystic fibrosis (nutrition)	Complementary—bedside BIA using CF-specific prediction equations or raw R and Xc values.	Updated ESPEN-ESPGHAN-ECFS guideline on nutrition care in cystic fibrosis across all ages (86 recommendations, 100% agreement), emphasizing body composition over BMI alone because of its stronger association with pulmonary function and covering the shift toward healthier eating with CFTR modulators. Recommendation 6 (100% consensus) states that BIA may be considered a non-invasive bedside method for body composition in cystic fibrosis, given the limitations imposed by electrolyte imbalances and the lack of adult-specific equations.
Erdelyi, A., et al. (2024) [130]	Narrative review—Women’s health/menopause (nutrition)	Complementary—BIA mentioned only briefly as helpful for assessing nutritional status and body composition.	Review of nutrition in perimenopause and menopause, when cardiometabolic and osteoporosis risk rise with estrogen decline and basal metabolic rate falls by 250–300 kcal/day. Recommends energy adjustment, adequate protein, calcium and vitamin D, a Mediterranean-style diet and dietitian-led counseling.
Son, J. W., et al. (2025) [131]	Narrative review—Body composition methodology (BIA)	Primary—review dedicated to BIA technology and applications. Impedance (Z), composed of R and Xc, estimates TBW and hence FFM and FM.	Review of body-composition technology in obesity, sarcopenia and metabolic disease. PhA at 50 kHz indexes cellular integrity, hydration and muscle quality; BIVA plots height-normalized R/Xc to track hydration and detect hypo- or hyperhydration. Technology has moved from SF-BIA to MF-BIA and BIS, with octapolar systems for segmental analysis, while wearables and home scales use SF-BIA with limited accuracy. BIA is endorsed by EWGSOP and AWGS for sarcopenia; accuracy is good up to BMI 34 but falls in severe obesity (fat underestimated >30%), critical illness and across ethnicities without specific equations.
Weng, S.-E., et al. (2025) [132]	Systematic review and meta-analysis—Sarcopenia (epidemiology)	Complementary—BIA one of the diagnostic modalities compared in the subgroup analysis.	Review of 140 studies (156,325 Asian adults ≥60 years, AWGS 2019 criteria): pooled prevalence 16.5% for sarcopenia, 28.7% for possible sarcopenia and 4.4% for severe sarcopenia, with regional differences and higher figures in type 2 diabetes (20.5%). Prevalence varied by diagnostic modality, from 17.2% with BIA-based combinations (7.5% in the most restrictive BIA subgroup) up to 20.8% with DXA plus strength and performance measures.
Anagnostou, D., et al. (2025) [133]	Narrative review—Sarcopenia (cardiovascular)	Complementary—BIA mentioned only briefly as one of the muscle-mass tools under the EWGSOP2/AWGS criteria.	Review of the bidirectional relationship between sarcopenia and cardiovascular disease in older patients, with shared mechanisms and worse outcomes in heart failure, TAVI, acute coronary syndromes and atrial fibrillation. Treatment centers on resistance training and nutritional optimization (protein 1.2–1.6 g/kg/day).
Wang, T., et al. (2025) [134]	Narrative review—Sarcopenia/cachexia (mechanisms)	Complementary—BIA for estimating lean body mass in sarcopenia and cachexia.	Review of the molecular mechanisms and therapies in sarcopenia and cachexia, highlighting shared and distinct pathways and the adipose-muscle crosstalk mediated by adipokines, myokines and noncoding RNAs, and surveying multimodal treatment from exercise to gene, stem-cell and microbiome therapies. BIA is suitable for ambulatory and bedridden patients but limited in accuracy.

## Appendix B

**Table A2:** Studies reporting primary data on bioelectrical impedance analysis (BIA) in clinical nutrition among most-cited publications (>20 cites/year) in the field, ordered by year of publication.

Authors	Type of Study	Study Area	BIA Usage	Summary of Main Findings
Bian, A., et al. (2020) [59]	Cross-sectional	Sarcopenia (endocrine/biomarkers)	Primary—body composition measured by BIA (InBody 720): ASMM index, percentage body fat, bone mineral content and body cell mass.	Study of 3276 Chinese adults ≥60 years diagnosed by AWGS criteria. Sarcopenia prevalence was 12.82% (11.10% in men, 17.03% in women) and increased with age. Serum GH, IGF-1, testosterone and MGF were all significantly lower in sarcopenic participants (*p* < 0.001). ASMI, measured by BIA, was significantly lower in the sarcopenia group; it correlated positively with GH, testosterone, IGF-1, MGF, gender, BMI, BUN, creatinine and hemoglobin, and negatively with HDL-C. IGF-1 and MGF, together with BMI and gender, were independently associated with ASMI, pointing to a key role in age-related muscle loss.
Bruci, A., et al. (2020) [135]	Prospective observational, real-life	Obesity (ketogenic diet/kidney)	Primary—bioimpedance the sole body-composition method, used to track FM, muscle mass and water compartments.	Study of 92 patients with obesity (38 with mild CKD, 54 with normal kidney function) following a VLCKD for approximately 3 months. Mean weight loss was ~20% of initial weight, ~80% of it FM, with only minor muscle loss. Blood pressure, fasting glucose, HbA1c, total cholesterol, triglycerides and uric acid improved, with no clinically relevant liver or kidney changes, and 27.7% of those with mild CKD normalized eGFR (≥90 mL/min/1.73 m^2^). No differences in efficacy or safety between groups, supporting VLCKD as safe and effective under medical supervision, including in mild kidney failure. TBW, ECW and ICW all fell significantly, consistent with the diuretic effect of ketosis.
Marini, E., et al. (2020) [89]	Cross-sectional	Sports/athletes	Primary—classic BIVA (R/H, Xc/H, Z/H), specific BIVA (Rsp, Xcsp, Zsp) and PhA compared to DXA (FM, FFM) and deuterium/bromide dilution (TBW, ECW, ICW).	Study of 202 athletes (139 men, 63 women) across 11 sports. Specific BIVA is preferable for evaluating %FM, classic BIVA for TBW, and both approaches detect ECW/ICW shifts through PhA, so the authors recommend using them complementarily for comprehensive body-composition monitoring in athletes. Further research is needed on different types of dehydration and on variation across the competitive season. Specific BIVA correlated strongly with FM and %FM but failed for TBW; classic BIVA correlated strongly and negatively with TBW but performed poorly for %FM (significant in men only). PhA correlated negatively with ECW/ICW in both sexes and positively with ICW in men. Body-geometry correction removed body-size differences versus reference populations.
Moro, T., et al. (2020) [136]	Randomized controlled trial (parallel-group)	Sports/athletes (nutrition)	Primary—BIA used solely to estimate FM and FFM.	Study in 16 elite under-23 cyclists allocated to time-restricted eating (8 h window, 10:00–18:00) or a normal diet for 4 weeks of pre-season training at identical intake (4800 kcal/day). TRE lowered body weight (−2%, *p* = 0.04) and improved the peak-power-to-weight ratio (*p* = 0.02) with no change in absolute performance, while free testosterone (−27%, *p* = 0.006) and IGF-1 (−12%, *p* = 0.03) decreased. The neutrophil-to-lymphocyte ratio fell only in TRE (*p* = 0.03), suggesting an anti-inflammatory, immune-protective effect. BIA showed a fall in FM (−1.1%, *p* = 0.01) with preserved FFM.
Kitamura, A., et al. (2021) [137]	Prospective cohort	Sarcopenia (outcomes/mortality)	Primary—ALST index measured by direct segmental MF-BIA (InBody 720), with cutoffs <7.0 kg/m^2^ (men) and <5.7 kg/m^2^ (women).	Study of 1851 Japanese residents ≥65 years followed for 5.8 years (AWGS 2019), with ALMI measured by direct segmental MF-BIA. Sarcopenia prevalence was 11.5% in men and 16.7% in women, reaching ~32% and ~48% at ≥80 years, and roughly doubled all-cause mortality and incident disability; severe sarcopenia roughly tripled both. Low ALMI alone, without low grip strength or slow gait, did not raise risk, supporting a diagnosis requiring both low muscle mass and impaired function.
Kim, N. H., et al. (2021) [138]	Cross-sectional	Obesity/body composition (WWI)	Complementary—BIA provided FM, SMM and %body fat alongside DXA and CT.	Study of 602 Korean older adults evaluating the weight-adjusted waist index (WWI). WWI rose with fat and fell with muscle measures, and the highest quartile showed more than three-fold odds of combined low muscle mass and high FM, suggesting it may identify sarcopenic obesity. BIA tracked the adiposity and muscle gradients of the index as closely as DXA did.
Beeri, M. S., et al. (2021) [139]	Prospective cohort	Sarcopenia (cognition/dementia)	Primary—BIA used solely to estimate SMM for a composite sarcopenia score combining muscle mass and grip strength.	Study within the Rush Memory and Aging Project: 1175 non-demented community-dwelling older adults (mean age 80.9 years; 77% women) followed a mean 5.6 years. More severe baseline sarcopenia predicted incident Alzheimer dementia, incident mild cognitive impairment and faster cognitive decline. Analyzed separately, muscle function (grip strength, gait speed) rather than lean mass drove these associations. Sarcopenia identifies older adults at risk of late-life cognitive impairment, but more granular muscle metrics are needed to improve predictive specificity.
Yee, X. S., et al. (2021) [140]	Cross-sectional	Sarcopenia (physical performance)	Primary—muscle mass measured by MF segmental BIA (TANITA MC-780M), yielding SMM index and FMI.	Study of 887 community-dwelling adults ≥50 years comparing sit-to-stand tests with grip strength. Sit-to-stand correlated weakly with grip strength but more strongly with gait speed, endurance and dynamic balance. Possible sarcopenia prevalence was similar across tests, whereas confirmed sarcopenia was lower with sit-to-stand than with grip strength. BIA-derived SMM correlated with grip strength but not with sit-to-stand, and higher FMI—not SMM index—predicted poorer sit-to-stand performance.
Konishi, M., et al. (2021) [141]	Multicenter prospective cohort, post hoc analysis	Sarcopenia (heart failure)	Primary—ASMM index measured by BIA as the muscle-mass component of the AWGS criteria.	Analysis of the FRAGILE-HF cohort: 942 hospitalized patients ≥65 years with heart failure. HFpEF patients showed higher rates of low handgrip strength and slow gait speed, producing similar overall sarcopenia prevalence. Sarcopenia independently predicted 1-year mortality in both HFpEF and HFrEF, with no interaction by ejection–fraction phenotype. Low ASMM by BIA was less frequent in HFpEF than in HFrEF.
Cheng, K. Y.-K., et al. (2021) [142]	Cross-sectional validation	Sarcopenia (BIA validation)	Primary—BIA (InBody 120 and 720) cross-validated against DXA for ASMM.	Study of 1587 Hong Kong community-dwelling adults (mean age 72 ± 12 years) cross-validating BIA against DXA for ASMM under AWGS 2019 criteria. BIA systematically overestimated the ASMM index despite strong correlation, the bias being larger in men and in obesity and yielding falsely low sarcopenia prevalence with raw cutoffs. Device-specific prediction equations (including sex and BMI) cut the difference to −0.02 ± 0.31 kg/m^2^; after adjustment, prevalence in the ≥65 subgroup was 39.4–40.8%. With population- and device-specific correction BIA works as a quick screening tool where DXA is unavailable.
Merchant, R. A., et al. (2021) [143]	Cross-sectional	Frailty/sarcopenia (body composition)	Primary—body composition by MF-BIA (InBody S10): FMI, FFMI, FM/FFM ratio, PhA and body cell mass.	Study of 191 pre-frail older adults (mean age 73.0 ± 5.7 years; 57.1% female) relating BMI, FMI, FFMI and the FM/FFM ratio to physical function, cognition and sarcopenia. Higher FFMI and lower FM/FFM, particularly in the high-BMI group, were associated with better handgrip strength, gait speed, SPPB and MoCA scores and with lower odds of sarcopenia, while the highest FMI and FM/FFM tertiles showed more sarcopenic obesity; low muscle mass was more prevalent in the normal-BMI group. FFMI and FM/FFM predicted functional outcomes better than BMI, so interventions should focus on increasing FFMI. PhA was significantly higher in the lowest FMI and FM/FFM tertiles and in the highest FFMI tertile, with body cell mass following the same pattern.
Davis, J. N., et al. (2021) [144]	Cluster randomized controlled trial	Pediatric nutrition/obesity (intervention)	Complementary—BIA used only to measure body fat percentage as one anthropometric outcome.	Study of the Texas Sprouts gardening, nutrition and cooking program in 16 elementary schools (3135 children). Vegetable intake increased significantly after one school year, but there was no effect on adiposity, BMI parameters, waist circumference or blood pressure.
Lee, Y. S., et al. (2021) [145]	Retrospective multicenter, model development and validation	Body composition methodology (AI/imaging)	Complementary—BIA-derived ALST and FM as the clinical reference for validating the CT model.	Study developing and validating a 3D U-Net deep neural network for automatic volumetric body-composition segmentation on whole-body PET-CT, with external validation in three datasets and an elderly cohort. Waist muscle and fat areas were independently associated with sarcopenia and dysmobility syndrome. CT muscle measures tracked BIA-derived ALST and CT fat measures tracked BIA FM, and the proposed CT cutoffs were anchored to BIA thresholds for low ALST and high FM.
Akamatsu, Y., et al. (2022) [146]	Cross-sectional	Sarcopenia (PhA/muscle quality)	Primary—PhA and body composition measured by BIA.	Study of 1419 Japanese adults testing PhA as a marker of muscle quality. Mean PhA fell with age (young men 6.25 vs. older men 5.26°; young women 5.37 vs. older women 4.61) and was independently associated with SMM index and muscle quality (grip strength/upper-limb muscle mass) in both sexes. PhA was significantly lower in sarcopenia among young men, young women and older women, with the same trend but no significant difference in older men. ROC accuracy was good: AUC 0.882 (cutoff 5.95°) in young men, 0.838 (5.04) in older men, 0.865 (5.02) in young women and 0.850 (4.20) in older women. PhA reflects muscle quality beyond muscle mass, provided age- and sex-specific references are applied.
Petermann–Rocha, F., et al. (2022) [147]	Prospective cohort	NAFLD/liver (muscle mass)	Primary—SMM estimated by BIA (Tanita BC418MA) via the Janssen equation and adjusted for weight.	Study of 333,295 UK Biobank participants with a median 10-year follow-up examining muscle mass, estimated by BIA with the Janssen equation and adjusted for body weight, and grip strength (Jamar dynamometer) as predictors of incident severe NAFLD. Compared with the lowest tertile, the highest tertiles of muscle mass (HR 0.46) and grip strength (HR 0.70) showed significantly lower risk. The muscle mass-NAFLD association was non-linear (*p* < 0.001) whereas grip strength was linear. Assuming causality, 33.1% and 17.7% of severe NAFLD cases were attributable to low muscle mass and low grip strength, respectively, suggesting that improving physical capability may be protective and warranting intervention trials.
Anand, S. S., et al. (2022) [60]	Cross-sectional	Obesity/adiposity (cognition)	Complementary—BIA used only to measure body fat percentage.	Analysis of 9189 adults free of cardiovascular disease (CAHHM and PURE-MIND). Higher body fat and visceral adipose tissue were independently associated with lower DSST cognitive scores, each 1-SD increase equating to ~1 year of cognitive aging, with no association for MoCA. BIA-derived body fat percentage correlated closely with MRI-derived visceral adipose tissue.
Wang, Y., et al. (2022) [148]	Observational, metagenomics	Sarcopenia (gut microbiome)	Primary—ASMM measured by direct segmental MF-BIA (InBody 770) for sarcopenia diagnosis under AWGS 2019 criteria, used only as a measurement tool.	Study of 1417 rural Chinese middle-aged and older adults (sarcopenia prevalence 10%) using shotgun metagenomics. Beta-diversity differed between sarcopenic and non-sarcopenic subjects while alpha-diversity did not. Seven species were enriched in sarcopenia, six of them tracking severity, and a species-based random-forest classifier reached AUC 0.852.
Tang, T., et al. (2022) [149]	Prospective cohort	Oncology (sarcopenia biomarkers)	Primary—SMM estimated by BIA (InBody 770) as part of the AWGS 2019 reference standard for sarcopenia.	Study of 579 patients with advanced NSCLC (derivation and validation sets) evaluating the serum creatinine/cystatin C ratio (CCR) and sarcopenia index (SI) as surrogate diagnostic markers of sarcopenia. Both markers correlated with handgrip strength, and CCR-, SI- and AWGS-defined sarcopenia each independently predicted mortality (HR 1.55–1.76). The two indices show comparable accuracy and may serve as inexpensive surrogate biomarkers in cancer patients, with CCR possibly better at predicting overall survival, pending external validation. CCR and SI correlated positively with both BIA- and CT-derived muscle mass.
Otsuka, Y., et al. (2022) [150]	Randomized controlled trial (single-blind, three-arm parallel-group)	Exercise/sarcopenia (resistance training)	Primary—segmental BIS (S-BIS) used to assess muscle quantity and quality.	24-week trial in 50 adults aged 50–79 comparing no exercise with low- and moderate-intensity resistance training, assessed by MRI, DXA and segmental BIS. Moderate intensity improved muscle quantity and quality, with detected changes in the intracellular resistance index, membrane capacitance and PhA. Low intensity improved quantity only. Changes in PhA, capacitance and resistance ratio tracked thigh CSA but not subcutaneous fat, and the intracellular resistance index was more sensitive than DXA leg lean mass.
Asvold, B. O., et al. (2023) [151]	Cohort profile	Population health (cohort profile)	Complementary—BIA (InBody 770) added in HUNT4-N as a new measurement tool.	Cohort profile update for the HUNT Study (Norway), with repeated surveys since 1984–86 and linkage to health registries; HUNT4 (2017–19) included 56,042 participants in Nord-Trondelag and 107,711 in Sor-Trondelag. New measures include body composition, 1-week accelerometry, physical and cognitive testing in older adults and genotyping. BIA was applied to nearly all clinical-examination participants able to stand barefoot.
Jeong, S.-M., et al. (2023) [152]	Cross-sectional	Obesity (BMI diagnosis)	Marginal use—adiposity is assessed with DXA as the reference method; BIA data appear only as figures measured by other authors and cited for comparison.	Study using nationally representative data from US NHANES (1999–2006; n = 18,061) and Korea NHANES (2008–2011; n = 18,861) to test how well BMI reflects body fatness. BMI correlated strongly with FMI and truncal fat but less well with percentage body fat; correlations were stronger in women, declined with age and were markedly lower in Koreans than in Whites, Blacks or Mexican–Americans. Among obesity-related biomarkers, insulin correlated most strongly with adiposity indices, and BMI predicted biomarkers comparably to FMI and better than percentage body fat. BMI is a valid adiposity proxy, particularly in younger adults, women and Western populations, but requires cautious interpretation in older adults and Asian populations.
Travers, J., et al. (2023) [153]	Randomized controlled trial (multicenter)	Frailty (intervention)	Complementary—BIA for secondary body-composition outcomes.	Study in six Irish primary-care practices: 168 community-dwelling adults ≥65 years randomized to a 3-month home-based strength-exercise program plus dietary protein or usual care, with 156 completing follow-up. Frailty was significantly reduced and grip strength improved. Of the BIA outcomes only bone mass changed significantly, while muscle mass, body fat and biological age favored the intervention without reaching significance. BIA absolute accuracy is reduced at high BMI, making it better suited to tracking change over time than to single absolute measurements.
Liu, C., et al. (2023) [154]	Cross-sectional	Sarcopenic obesity (body composition)	Primary— BIA is used as a body composition assessment tool.	Study of 1637 Chinese adults ≥60 years (AWGS 2019) with body composition by BIA (InBody 120): ASMM index and body fat percentage, plus a BIA prediction model for DXA-equivalent ASMM index. BMI-defined obesity was protective against sarcopenia whereas fat-percentage-defined obesity was a risk factor, U-shaped in women and linear in men. Recommended fat ranges to prevent both conditions: 26.0–34.6% in older women and <23.9% in older men. Appendicular fat mass percentage was inversely related to muscle mass and strength.
Kiss, N., et al. (2023) [155]	Prospective cohort	Oncology (sarcopenia/malnutrition)	Primary—ALST derived from SF-BIA (Tanita BC418MA) FFM through a prediction equation, then scaled by height^2^ and by BMI.	UK Biobank study of 4122 adults with cancer comparing two ALST scaling methods over a median 11.2 years (901 deaths). Low muscle mass, malnutrition and sarcopenia predicted all-cause and cancer-specific mortality under both methods, with stronger hazard ratios for height^2^ scaling. ALST/BMI identified far more cases than ALST/height^2^ (low muscle mass 8.0% vs. 1.7%; malnutrition 11.2% vs. 6.2%; sarcopenia 1.4% vs. 0.2%), especially with obesity, and overlap was minimal (0.3%). BMI adjustment is preferred in obesity, but reporting both methods is advisable.
Lin, X., et al. (2024) [156]	Experimental device development	Biosensors/wearable technology	Marginal use—restricted to the electrode–skin interface; ‘phase angle’ appears only in Bode plots from electrochemical impedance spectroscopy.	Study developing a wearable biosensor combining conductive MXene and porous c-MOF (Ni3(HITP)2) on non-woven fabric for muscle theranostics in hyperuricemia- and hyperglycemia-related lower-limb dysfunction. The sensor detected uric acid and glucose in artificial sweat with wireless transmission, and the MOF/MXene electrode showed lower electrode–skin impedance and higher signal-to-noise than Ag/AgCl for electrophysiological recording plus nearly double the charge storage capacity, enabling electrostimulation therapy. The phase angle referred to in this paper comes from Bode plots of the electrode–skin interface (impedance spectroscopy) and is not the BIA-derived PhA.
Feng, Q., et al. (2024) [157]	Prospective cohort	Obesity/adiposity (cardiovascular)	Primary—body fat percentage measured by SF-BIA (Tanita BC418MA).	Study of 468,333 UK Biobank participants over 12 years comparing waist-to-height ratio (WHtR) with body fat percentage for ischemic cardiovascular disease. The body fat association vanished after adjustment for WHtR (HR 1.20 to 0.99 per 5% increase), indicating central fat distribution drives risk; a sensitivity analysis in ~34,000 participants gave consistent null results for BIA- and DXA-derived body fat. WHtR was linearly and independently associated with ischemic CVD, myocardial infarction and stroke (HR ~1.22–1.24 per 5%), with ≥0.5 marking higher risk in both sexes.
Reddy, Y. N. V., et al. (2025) [158]	Cross-sectional analysis of a prospective cohort	Obesity/adiposity (heart failure)	Complementary—bioimpedance used only to estimate FM as one of several adiposity measures.	Analysis of the PVDOMICS study (276 HFpEF patients, replicated in an independent cohort of 254). Obesity, independently of insulin resistance or diabetes, was associated with greater biventricular remodeling, higher pulmonary capillary wedge pressure and worse functional capacity, challenging the concept of a distinct ‘diabetic’ HFpEF phenotype.

## References

[1] Schwan H.P. (1957). Electrical properties of tissue and cell suspensions. Adv. Biol. Med. Phys..

[2] Cole K.S., Cole R.H. (1941). Dispersion and absorption in dielectrics I. Alternating current characteristics. J. Chem. Phys..

[3] Thomasset A. (1962). Bioelectric properties of tissue. Impedance measurement in clinical medicine. Significance of curves obtained. Lyon Méd..

[4] Thomasset A. (1963). Bio-electric properties of tissues. Estimation by measurement of impedance of extracellular ionic strength and intracellular ionic strength in the clinic. Lyon Méd..

[5] Nyboer J. (1959). Electrical Impedance Plethysmography.

[6] Hoffer E.C., Meador C.K., Simpson D.C. (1969). Correlation of whole-body electrical impedance with total body water volume. J. Appl. Physiol..

[7] Lukaski H.C., Johnson P.E., Bolonchuk W.W., Lykken G.I. (1985). Assessment of fat-free mass using bioelectrical impedance measurement of the human body. Am. J. Clin. Nutr..

[8] Lukaski H.C., Bolonchuk W.W., Hall C.B., Siders W.A. (1986). Validation of tetrapolar bioelectrical impedance method to assess human body composition. J. Appl. Physiol..

[9] Kushner R.F., Schoeller D.A. (1986). Estimation of total body water by bioelectrical impedance analysis. Am. J. Clin. Nutr..

[10] Segal K.R., Van Loan M., Fitzgerald P.I., Hodgdon J.A., Van Itallie T.B. (1988). Lean body mass estimation by bioelectrical impedance analysis: A four-site cross-validation study. Am. J. Clin. Nutr..

[11] Deurenberg P., van der Kooy K., Leenen R., Weststrate J.A., Seidell J.C. (1991). Sex and age specific prediction formulas for estimating body composition from bioelectrical impedance: A cross-validation study. Int. J. Obes..

[12] National Institutes of Health (1996). Bioelectrical impedance analysis in body composition measurement: National Institutes of Health Technology Assessment Conference Statement. Am. J. Clin. Nutr..

[13] Sun S.S., Chumlea W.C., Heymsfield S.B., Lukaski H.C., Schoeller D.A., Friedl K.E., Kuczmarski R.J. (2003). Development of bioelectrical impedance analysis prediction equations for body composition using a multicomponent model for use in epidemiologic surveys. Am. J. Clin. Nutr..

[14] Ward L.C., Bunce I.H., Cornish B.H., Mirolo B.R., Thomas B.J., Jones L.C. (1992). Multi-frequency bioelectrical impedance augments the diagnosis and management of lymphoedema in post-mastectomy patients. Eur. J. Clin. Investig..

[15] Organ L.W., Bradham G.B., Gore D.T., Lozier S.L. (1994). Segmental bioelectrical impedance analysis: Theory and application of a new technique. J. Appl. Physiol..

[16] Brown B.H., Barber D.C., Seagar A.D. (1985). Applied potential tomography: Possible clinical applications. Clin. Phys. Physiol. Meas..

[17] Cheney M., Isaacson D., Newell J.C. (1999). Electrical impedance tomography. SIAM Rev..

[18] Borcea L. (2002). Electrical impedance tomography. Inverse Probl..

[19] Adler A., Holder D.S. (2021). Electrical Impedance Tomography: Methods, History and Applications.

[20] Bodenstein M., David M., Markstaller K. (2009). Principles of electrical impedance tomography and its clinical application. Crit. Care Med..

[21] De Lorenzo A., Andreoli A., Matthie J., Withers P. (1997). Predicting body cell mass using theoretical methods: A technological review. J. Appl. Physiol..

[22] Matthie J.R. (2008). Bioimpedance measurements of human body composition: Critical analysis and outlook. Expert Rev. Med. Devices.

[23] Piccoli A., Rossi B., Pillon L., Bucciante G. (1994). A new method for monitoring body fluid variation by bioelectrical impedance analysis: The RXc graph. Kidney Int..

[24] Piccoli A., Nigrelli S., Caberlotto A., Botazzo S., Rossi B., Pillon L., Maggiore Q., Bucciante G. (1995). Bivariate normal values of the bioelectrical impedance vector in adult and elderly populations. Am. J. Clin. Nutr..

[25] Norman K., Stobäus N., Pirlich M., Bosy-Westphal A. (2012). Bioelectrical phase angle and impedance vector analysis—Clinical relevance and applicability of impedance parameters. Clin. Nutr..

[26] Kyle U.G., Bosaeus I., De Lorenzo A.D., Deurenberg P., Elia M., Gómez J.M., Heitmann B.L., Kent-Smith L., Melchior J.C., Pirlich M. (2004). Bioelectrical impedance analysis—Part I: Review of principles and methods. Clin. Nutr..

[27] Kyle U.G., Bosaeus I., De Lorenzo A.D., Deurenberg P., Elia M., Gómez J.M., Heitmann B.L., Kent-Smith L., Melchior J.C., Pirlich M. (2004). Bioelectrical impedance analysis—Part II: Utilization in clinical practice. Clin. Nutr..

[28] Deurenberg P., Weststrate J.A., Seidell J.C. (1991). Body mass index as a measure of body fatness: Age- and sex-specific prediction formulas. Br. J. Nutr..

[29] Ellis K.J. (2000). Human body composition: In Vivo Methods. Physiol. Rev..

[30] Nana A., Slater G.J., Hopkins W.G., Burke L.M. (2012). Techniques for undertaking dual-energy X-ray absorptiometry whole-body scans to estimate body composition in tall and/or broad subjects. Int. J. Sport Nutr. Exerc. Metab..

[31] Wang L.-C., Raimann J.G., Tao X., Preciado P., Thwin O., Rosales L., Thijssen S., Kotanko P., Zhu F. (2022). Estimation of fluid status using three multifrequency bioimpedance methods in hemodialysis patients. Hemodial. Int..

[32] National Center for Health Statistics (NCHS) (2002). National Health and Nutrition Examination Survey: NHANES 1999–2000.

[33] Baumgartner C., Hasgall P.A., Di Gennaro F., Neufeld E., Lloyd B., Gosselin M.C., Payne D., Klingenböck A., Kuster N. (2025). IT’IS Database for Thermal and Electromagnetic Parameters of Biological Tissues, v. 5.0. https://itis.swiss/virtual-population/tissue-properties/downloads/database-v5-0.

[34] Campa F., Coratella G., Cerullo G., Noriega Z., Francisco R., Charrier D., Irurtia A., Lukaski H., Silva A.M., Paoli A. (2024). High-standard predictive equations for estimating body composition using bioelectrical impedance analysis: A systematic review. J. Transl. Med..

[35] Kampo D., Závodná E., Vondra V. (2025). Multi-frequency bioimpedance analysis in practice: A review of validated prediction equations for key body composition parameters. Physiol. Res..

[36] Kanellakis S., Skoufas E., Karaglani E., Ziogos G., Koutroulaki A., Loukianou F., Michalopoulou M., Gkeka A., Marikou F., Manios Y. (2020). Development and validation of a bioelectrical impedance prediction equation estimating fat free mass in Greek—Caucasian adult population. Clin. Nutr. ESPEN.

[37] Wahrlich V., Ciafrino A., Chain A., Bossan F.M., Baltar V.T., dos Anjos L.A. (2026). Body composition measures assessed by bioelectrical impedance analysis and dual-energy X-ray absorptiometry in a sample of Brazilian adults and older adults. Front. Nutr..

[38] Lukaski H.C., Bolonchuk W.W. (1988). Estimation of body fluid volumes using tetrapolar bioelectrical impedance measurements. Aviat. Space Environ. Med..

[39] Kyle U.G., Genton L., Hans D., Pichard C. (2003). Validation of a bioelectrical impedance analysis equation to predict appendicular skeletal muscle mass (ASMM). Clin. Nutr..

[40] Hughes J.T., Maple-Brown L.J., Piers L.S., Meerkin J., O’Dea K., Ward L.C. (2015). Development of a single-frequency bioimpedance prediction equation for fat-free mass in an adult Indigenous Australian population. Eur. J. Clin. Nutr..

[41] Blake G.M., Fogelman I. (2007). The role of DXA bone density scans in the diagnosis and treatment of osteoporosis. Postgrad. Med. J..

[42] Marra M., Sammarco R., De Lorenzo A., Iellamo F., Siervo M., Pietrobelli A., Donini L.M., Santarpia L., Cataldi M., Pasanisi F. (2019). Assessment of body composition in health and disease using bioelectrical impedance analysis (BIA) and dual energy X-ray absorptiometry (DXA): A critical overview. Contrast Media Mol. Imaging.

[43] Shepherd J.A., Ng B.K., Sommer M.J., Heymsfield S.B. (2017). Body composition by DXA. Bone.

[44] Pietrobelli A., Formica C., Wang Z., Heymsfield S.B. (1996). Dual-energy X-ray absorptiometry body composition model: Review of physical concepts. Am. J. Physiol..

[45] Heymsfield S.B., Ebbeling C.B., Zheng J., Pietrobelli A., Strauss B.J., Silva A.M., Ludwig D.S. (2015). Multi-component molecular-level body composition reference methods: Evolving concepts and future directions. Obes. Rev..

[46] Hind K., Oldroyd B., Truscott J. (2011). In vivo precision of the GE Lunar iDXA densitometer for the measurement of total body composition and fat distribution in adults. Eur. J. Clin. Nutr..

[47] Park S.S., Lim S., Kim H., Kim K.M. (2021). Comparison of two DXA systems, Hologic Horizon W and GE Lunar Prodigy, for assessing body composition in healthy Korean adults. Endocrinol. Metab..

[48] Siekman B., Gagliardi A., Gantz M., Williams K., Tinsley G.M., Heymsfield S.B., Ramirez S., Thomas D.M. (2026). Conversion of DXA body composition measurements across different devices: A standardized approach. Eur. J. Clin. Nutr..

[49] Kim S., Lee G.Y., Yoo J.I., Lee S.W., Ha Y.C. (2026). Cross-calibration of bone mineral density and body composition between GE Lunar Prodigy and Hologic Horizon W dual energy X-ray absorptiometry systems. J. Bone Metab..

[50] Prado C.M., Gonzalez M.C., Norman K., Barazzoni R., Cederholm T., Compher C., Jensen G.L., Abe T., Barbosa-Silva T.G., Bosy-Westphal A. (2025). Methodological standards for body composition-an expert-endorsed guide for research and clinical applications: Levels, models, and terminology. Am. J. Clin. Nutr..

[51] Kaul S., Rothney M.P., Peters D.M., Wacker W.K., Davis C.E., Sever P.J., Ergun D.L. (2012). Dual-energy X-ray absorptiometry for quantification of visceral fat. Obesity.

[52] Borga M., West J., Bell J.D., Harvey N.C., Romu T., Heymsfield S.B., Dahlqvist Leinhard O. (2018). Advanced body composition assessment: From body mass index to body composition profiling. J. Investig. Med..

[53] De Paula E Mancilha T., Bolan P.J., Dengel D.R., Oldenburg N., Taddese A., Yentzer B.P., Lysne L., Wong A., LaPage N., Wang Q. (2026). Visceral adipose tissue in obesity: A comparison between DXA and MRI measures. J. Clin. Densitom..

[54] Sheean P., Gonzalez M.C., Prado C.M., McKeever L., Hall A.M., Braunschweig C.A. (2020). American Society for Parenteral and Enteral Nutrition clinical guidelines: The validity of body composition assessment in clinical populations. JPEN J. Parenter. Enter. Nutr..

[55] Basty N., Thanaj M., Whitcher B., Bell J.D., Thomas E.L. (2026). Comparing DXA and MRI body composition measurements in cross-sectional and longitudinal cohorts. Commun. Med..

[56] Achamrah N., Colange G., Delay J., Rimbert A., Folope V., Petit A., Grigioni S., Déchelotte P., Coëffier M. (2018). Comparison of body composition assessment by DXA and BIA according to the body mass index: A retrospective study on 3655 measures. PLoS ONE.

[57] Farias F., Severo M., Ramos E., Lopes C., Nogueira L., Araújo J. (2026). Assessment of fat mass and fat-free mass in Portuguese adults: Calibration of single-frequency leg-to-leg bioelectrical impedance with dual-energy x-ray absorptiometry. Nutrition.

[58] Zaplatosch M.E., Meireles J.F., Amason J.S., Dabeer S., Kliszczewicz B.M., Mangine G.T., Barry V.G., Gower B.A., Ingram K.H. (2025). Validity of body composition estimates in women assessed by a multifrequency bioelectrical impedance device. Sensors.

[59] Bian A., Ma Y., Zhou X., Guo Y., Wang W., Zhang Y., Wang X. (2020). Association between sarcopenia and levels of growth hormone and insulin-like growth factor-1 in the elderly. BMC Musculoskelet. Disord..

[60] Anand S.S., Friedrich M.G., Lee D.S., Awadalla P., Després J.P., Desai D., de Souza R.J., Dummer T., Parraga G., Larose E. (2022). Evaluation of Adiposity and Cognitive Function in Adults. JAMA Netw. Open.

[61] Ponti F., Santoro A., Mercatelli D., Gasperini C., Conte M., Martucci M., Sangiorgi L., Franceschi C., Bazzocchi A. (2020). Aging and Imaging Assessment of Body Composition: From Fat to Facts. Front. Endocrinol..

[62] Schwan H.P. (1992). Linear and nonlinear electrode polarization and biological materials. Ann. Biomed. Eng..

[63] Geddes L.A. (1997). Historical evolution of circuit models for the electrode–electrolyte interface. Ann. Biomed. Eng..

[64] Martinsen Ø.G., Heiskanen A. (2023). Bioimpedance and Bioelectricity Basics.

[65] Franks W., Schenker I., Schmutz P., Hierlemann A. (2005). Impedance characterization and modeling of electrodes for biomedical applications. IEEE Trans. Biomed. Eng..

[66] Ben Ishai P., Talary M.S., Caduff A., Levy E., Feldman Y. (2013). Electrode polarization in dielectric measurements: A review. Meas. Sci. Technol..

[67] Tjon K.C.E., Yuan J. (2019). Impedance characterization of silver/silver chloride micro-electrodes for bio-sensing applications. Electrochim. Acta.

[68] Dutra D., Bertemes-Filho P. (2018). Extracting parasite effects of electrical bioimpedance measurements. J. Electr. Bioimpedance.

[69] Liu T., Hu X., Wang D., Shi W., Xing D., You C., Xu T., Tian M., Qu L., Zhang X. (2025). Flexible dry electrodes for electrocardiographic monitoring: Working principles, design strategies, and recent advances. iScience.

[70] Park K., Jeong H., Jung Y., Suh J.-H., Je M., Kim J. (2025). Using biopotential and bio-impedance for intuitive human–robot interaction. Nat. Rev. Electr. Eng..

[71] Fu Y., Zhao J., Dong Y., Wang X. (2020). Dry Electrodes for Human Bioelectrical Signal Monitoring. Sensors.

[72] Kusche R., Kaufmann S., Ryschka M. (2019). Dry electrodes for bioimpedance measurements—Design, characterization and comparison. Biomed. Phys. Eng. Express.

[73] Levit B., Krebsbach P., Bar-Haim C., Hernandez-Sosa G., Hanein Y. (2023). Printed soft skin electrodes for seamless bio-impedance measurements. Flex. Print. Electron..

[74] Jeong I., Chung W.G., Kim E., Park W., Song H., Lee J., Oh M., Kim E., Paek J., Lee T. (2025). Machine learning in biosignal analysis from wearable devices. Mater. Horiz..

[75] Arabsalmani N., Ghouchani A., Jafarabadi Ashtiani S., Zamani M. (2025). Exploring Bio-Impedance Sensing for Intelligent Wearable Devices. Bioengineering.

[76] Groenendaal W., Lee S., van Hoof C. (2021). Wearable bioimpedance monitoring: Viewpoint for application in chronic conditions. JMIR Biomed. Eng..

[77] Chen J., Yin J., Liu Y., Kulkarni N., Saito E., Saucedo B.J., Young M., Chen J. (2026). Advances in Wearable Bioimaging. Adv. Mater..

[78] Luo Y., Abiri P., Zhang S., Chang C.C., Kaboodrangi A.H., Li R., Sahib A.K., Bui A., Kumar R., Woo M. (2018). Non-Invasive Electrical Impedance Tomography for Multi-Scale Detection of Liver Fat Content. Theranostics.

[79] Frerichs I., Amato M.B.P., van Kaam A.H., Tingay D.G., Zhao Z., Grychtol B., Bodenstein M., Gagnon H., Böhm S.H., Teschner E. (2017). Chest electrical impedance tomography examination, data analysis, terminology, clinical use and recommendations: Consensus statement of the TRanslational EIT developmeNt stuDy group. Thorax.

[80] Youssef Baby L., Bedran R.S., Doumit A., El Hassan R.H., Maalouf N. (2024). Past, present, and future of electrical impedance tomography and myography for medical applications: A scoping review. Front. Bioeng. Biotechnol..

[81] Frerichs I., Scaramuzzo G., Jonkman A. (2026). Recent advances in experimental and clinical applications of chest electrical impedance tomography: A narrative review. Intensive Care Med. Exp..

[82] Sonbas-Cobb B., Rutkove S.B., Wei B., Robicheau S., Arnold W.D., Hives K., Bartlett A., Riley P., Pagan A., Chrzanowski S.M. (2026). Multifrequency Electrical Impedance Myography Enhanced with Machine Learning for Screening Patients with Neuromuscular Disorders. Ann. Biomed. Eng..

[83] Barbosa-Silva M.C., Barros A.J., Wang J., Heymsfield S.B., Pierson R.N. (2005). Bioelectrical impedance analysis: Population reference values for phase angle by age and sex. Am. J. Clin. Nutr..

[84] Adami G.F., Marinari G., Gandolfo P., Cocchi F., Friedman D., Scopinaro  N. (1993). The use of bioelectrical impedance analysis for monitoring body composition changes during nutritional support. Surg Today.

[85] Sukkar S.G., Giacosa A., Frascio F. (1993). Validazione clinica della impedenziometria bioelettrica (BIA) nel paziente oncologico malnutrito [Clinical validation of bioelectrical impedance analysis (BIA) in malnourished cancer patients]. Riv. Ital. Nutr. Parenter. Enterale.

[86] Toso S., Piccoli A., Gusella M., Menon D., Bononi A., Crepaldi G., Ferrazzi E. (2000). Altered tissue electric properties in lung cancer patients as detected by bioelectric impedance vector analysis. Nutrition.

[87] Gupta D., Lis C.G., Dahlk S.L., Vashi P.G., Grutsch J.F., Lammersfeld C.A. (2004). Bioelectrical impedance phase angle as a prognostic indicator in advanced pancreatic cancer. Br. J. Nutr..

[88] Mattiello R., Amaral M.A., Mundstock E., Ziegelmann P.K. (2020). Reference values for the phase angle of the electrical bioimpedance: Systematic review and meta-analysis involving more than 250,000 subjects. Clin. Nutr..

[89] Marini E., Campa F., Buffa R., Stagi S., Matias C.N., Toselli S., Sardinha L.B., Silva A.M. (2020). Phase angle and bioelectrical impedance vector analysis in the evaluation of body composition in athletes. Clin. Nutr..

[90] Uemura K., Doi T., Tsutsumimoto K., Nakakubo S., Kim M.J., Kurita S., Ishii H., Shimada H. (2020). Predictivity of bioimpedance phase angle for incident disability in older adults. J. Cachexia Sarcopenia Muscle.

[91] de Borba E.L., Ceolin J., Ziegelmann P.K., Bodanese L.C., Gonçalves M.R., Cañon-Montañez W., Mattiello R. (2022). Phase angle of bioimpedance at 50 kHz is associated with cardiovascular diseases: Systematic review and meta-analysis. Eur. J. Clin. Nutr..

[92] Lukaski H.C., Talluri A. (2023). Phase angle as an index of physiological status: Validating bioelectrical assessments of hydration and cell mass in health and disease. Rev. Endocr. Metab. Disord..

[93] Bellido D., García-García C., Talluri A., Lukaski H.C., García-Almeida J.M. (2023). Future lines of research on phase angle: Strengths and limitations. Rev. Endocr. Metab. Disord..

[94] Hirata K., Yamada Y., Iida N., Kanda A., Shoji M., Yoshida T., Akagi R. (2023). Relation of leg phase angle from bioelectrical impedance analysis with voluntary and evoked contractile properties of the plantar flexors. Front. Physiol..

[95] Guerrini A., Siotto M., Germanotta M., Cipollini V., Cortellini L., Pavan A., Insalaco S., Khazrai Y.M., Aprile I. (2024). Muscle quality improvement in subacute post-stroke patients after rehabilitation: Usefulness of segmental phase angle from bioelectrical impedance analysis. Clin. Nutr..

[96] Asano Y., Tsunoda K., Nagata K., Lim N., Tsuji T., Shibuya K., Okura T. (2025). Segmental phase angle and the extracellular to intracellular water ratio are associated with functional disability in community-dwelling older adults: A follow-up study of up to 12 years. Nutrition.

[97] Guerrini A., Siotto M., Cocco C., Germanotta M., Cipollini V., Cortellini L., Pavan A., Lattanzi S., Insalaco S., Khazrai Y.M. (2025). Usefulness of body composition assessment by bioelectrical impedance vector analysis in subacute post-stroke patients in rehabilitation. Sci. Rep..

[98] Buffa R., Saragat B., Cabras S., Rinaldi A.C., Marini E. (2013). Accuracy of specific BIVA for the assessment of body composition in the United States population. PLoS ONE.

[99] Marini E., Buffa R., Saragat B., Coin A., Toffanello E.D., Berton L., Manzato E., Sergi G. (2012). The potential of classic and specific bioelectrical impedance vector analysis for the assessment of sarcopenia and sarcopenic obesity. Clin. Interv. Aging.

[100] Rosa G.B., Lukaski H.C., Sardinha L.B. (2025). The science of bioelectrical impedance-derived phase angle: Insights from body composition in youth. Rev. Endocr. Metab. Disord..

[101] Rosa G.B., Francisco R., Silva A.M., Lukaski H.C., Sardinha L.B. (2026). Changes in raw bioelectrical impedance parameters as markers of body composition in youth: A prospective cohort investigation. Nutrition.

[102] Bora D.J., Manikonda S.K.G. (2026). Applications of bioimpedance measurement in clinical and allied fields: A comprehensive review. Biomed. Eng. Online.

[103] Chen L.-K., Woo J., Assantachai P., Auyeung T.-W., Chou M.-Y., Iijima K., Jang H.C., Kang L., Kim M., Kim S. (2020). Asian Working Group for Sarcopenia: 2019 Consensus Update on Sarcopenia Diagnosis and Treatment. J. Am. Med. Dir. Assoc..

[104] Walowski C.O., Braun W., Maisch M.J., Jensen B., Peine S., Norman K., Mueller M.J., Bosy-Westphal A. (2020). Reference Values for Skeletal Muscle Mass—Current Concepts and Methodological Considerations. Nutrients.

[105] Perez-Campos Mayoral L., Mayoral Andrade G., Perez-Campos Mayoral E., Hernandez Hiterta T., Pina Canseco S., Rodal Canales F.J., Alejandro Cabrera-Fuentes H., Martinez Cruz M., Perez Santiago A.D., Jose Alpuche J. (2020). Obesity subtypes, related biomarkers & heterogeneity. Indian J. Med. Res..

[106] Ikizler T.A., Burrowes J.D., Byham-Gray L.D., Campbell K.L., Carrero J.-J., Chan W., Fouque D., Friedman A.N., Ghaddar S., Goldstein-Fuchs D.J. (2020). KDOQI Clinical Practice Guideline for Nutrition in CKD: 2020 Update. Am. J. Kidney Dis..

[107] Holmes C.J., Racette S.B. (2021). The Utility of Body Composition Assessment in Nutrition and Clinical Practice: An Overview of Current Methodology. Nutrients.

[108] Campa F., Toselli S., Mazzilli M., Gobbo L.A., Coratella G. (2021). Assessment of Body Composition in Athletes: A Narrative Review of Available Methods with Special Reference to Quantitative and Qualitative Bioimpedance Analysis. Nutrients.

[109] Di Vincenzo O., Marra M., Di Gregorio A., Pasanisi F., Scalfi L. (2021). Bioelectrical impedance analysis (BIA)-derived phase angle in sarcopenia: A systematic review. Clin. Nutr..

[110] Kasper A.M., Langan-Evans C., Hudson J.F., Brownlee T.E., Harper L.D., Naughton R.J., Morton J.P., Close G.L. (2021). Come Back Skinfolds, All Is Forgiven: A Narrative Review of the Efficacy of Common Body Composition Methods in Applied Sports Practice. Nutrients.

[111] Moonen H.P.F.X., Van Zanten A.R.H. (2021). Bioelectric impedance analysis for body composition measurement and other potential clinical applications in critical illness. Curr. Opin. Crit. Care.

[112] Lukaski H., Raymond-Pope C.J. (2021). New Frontiers of Body Composition in Sport. Int. J. Sports Med..

[113] Aprile G., Basile D., Giaretta R., Schiavo G., La Verde N., Corradi E., Monge T., Agustoni F., Stragliotto S. (2021). The Clinical Value of Nutritional Care before and during Active Cancer Treatment. Nutrients.

[114] Bossi P., Delrio P., Mascheroni A., Zanetti M. (2021). The Spectrum of Malnutrition/Cachexia/Sarcopenia in Oncology According to Different Cancer Types and Settings: A Narrative Review. Nutrients.

[115] Petermann-Rocha F., Balntzi V., Gray S.R., Lara J., Ho F.K., Pell J.P., Celis-Morales C. (2022). Global prevalence of sarcopenia and severe sarcopenia: A systematic review and meta-analysis. J. Cachexia Sarcopenia Muscle.

[116] Barazzoni R., Jensen G.L., Correia M.I.T.D., Gonzalez M.C., Higashiguchi T., Shi H.P., Bischoff S.C., Boirie Y., Carrasco F., Cruz-Jentoft A. (2022). Guidance for assessment of the muscle mass phenotypic criterion for the Global Leadership Initiative on Malnutrition (GLIM) diagnosis of malnutrition. Clin. Nutr..

[117] Shu X., Lin T., Wang H., Zhao Y., Jiang T., Peng X., Yue J. (2022). Diagnosis, prevalence, and mortality of sarcopenia in dialysis patients: A systematic review and meta-analysis. J. Cachexia Sarcopenia Muscle.

[118] Ackermans L.L.G.C., Rabou J., Basrai M., Schweinlin A., Bischoff S.C., Cussenot O., Cancel-Tassin G., Renken R.J., Gomez E., Sanchez-Gonzalez P. (2022). Screening, diagnosis and monitoring of sarcopenia: When to use which tool?. Clin. Nutr. ESPEN.

[119] Campa F., Gobbo L.A., Stagi S., Cyrino L.T., Toselli S., Marini E., Coratella G. (2022). Bioelectrical impedance analysis versus reference methods in the assessment of body composition in athletes. Eur. J. Appl. Physiol..

[120] Prado C.M., Landi F., Chew S.T.H., Atherton P.J., Molinger J., Ruck T., Gonzalez M.C. (2022). Advances in muscle health and nutrition: A toolkit for healthcare professionals. Clin. Nutr..

[121] Seron-Arbeloa C., Labarta-Monzon L., Puzo-Foncillas J., Mallor-Bonet T., Lafita-Lopez A., Bueno-Vidales N., Montoro-Huguet M. (2022). Malnutrition Screening and Assessment. Nutrients.

[122] Branco M.G., Mateus C., Capelas M.L., Pimenta N., Santos T., Makitie A., Ganhao-Arranhado S., Trabulo C., Ravasco P. (2023). Bioelectrical Impedance Analysis (BIA) for the Assessment of Body Composition in Oncology: A Scoping Review. Nutrients.

[123] Baek J.Y., Jung H.-W., Kim K.M., Kim M., Park C.Y., Lee K.-P., Lee S.Y., Jang I.-Y., Jeon O.H., Lim J.Y. (2023). Korean Working Group on Sarcopenia Guideline: Expert Consensus on Sarcopenia Screening and Diagnosis by the Korean Society of Sarcopenia, the Korean Society for Bone and Mineral Research, and the Korean Geriatrics Society. Ann. Geriatr. Med. Res..

[124] Cappellari G.G., Guillet C., Poggiogalle E., Pomar M.D.B., Batsis J.A., Boirie Y., Breton I., Frara S., Genton L., Gepner Y. (2023). Sarcopenic obesity research perspectives outlined by the sarcopenic obesity global leadership initiative (SOGLI). Clin. Nutr..

[125] Sweatt K., Garvey W.T., Martins C. (2024). Strengths and Limitations of BMI in the Diagnosis of Obesity: What is the Path Forward?. Curr. Obes. Rep..

[126] Liu D., Wang S., Liu S., Wang Q., Che X., Wu G. (2024). Frontiers in sarcopenia: Advancements in diagnostics, molecular mechanisms, and therapeutic strategies. Mol. Asp. Med..

[127] Voulgaridou G., Tyrovolas S., Detopoulou P., Tsoumana D., Drakaki M., Apostolou T., Chatziprodromidou I.P., Papandreou D., Giaginis C., Papadopoulou S.K. (2024). Diagnostic Criteria and Measurement Techniques of Sarcopenia: A Critical Evaluation of the Up-to-Date Evidence. Nutrients.

[128] Pereira J.P.D.C., Reboucas A.D.S., Prado C.M., Gonzalez M.C., Cabral P.C., Diniz A.D.S., Fayh A.P.T., Silva F.M. (2024). Phase angle as a marker of muscle quality: A systematic review and meta-analysis. Clin. Nutr..

[129] Wilschanski M., Munck A., Carrion E., Cipolli M., Collins S., Colombo C., Declercq D., Hatziagorou E., Hulst J., Kalnins D. (2024). ESPEN-ESPGHAN-ECFS guideline on nutrition care for cystic fibrosis. Clin. Nutr..

[130] Erdelyi A., Palfi E., Tuu L., Nas K., Szucs Z., Torok M., Jakab A., Varbiro S. (2024). The Importance of Nutrition in Menopause and Perimenopause—A Review. Nutrients.

[131] Son J.W., Han B.-D., Bennett J.P., Heymsfield S., Lim S. (2025). Development and clinical application of bioelectrical impedance analysis method for body composition assessment. Obes. Rev..

[132] Weng S.-E., Huang Y.-W., Tseng Y.-C., Peng H.-R., Lai H.-Y., Akishita M., Arai H., Hsiao F.-Y., Chen L.-K. (2025). The Evolving Landscape of Sarcopenia in Asia: A Systematic review and meta-analysis following the 2019 AWGS diagnostic criteria. Arch. Gerontol. Geriatr..

[133] Anagnostou D., Theodorakis N., Hitas C., Kreouzi M., Pantos I., Vamvakou G., Nikolaou M. (2025). Sarcopenia and Cardiogeriatrics: The Links Between Skeletal Muscle Decline and Cardiovascular Aging. Nutrients.

[134] Wang T., Zhou D., Hong Z. (2025). Sarcopenia and cachexia: Molecular mechanisms and therapeutic interventions. MedComm.

[135] Bruci A., Tuccinardi D., Tozzi R., Balena A., Santucci S., Frontani R., Mariani S., Basciani S., Spera G., Gnessi L. (2020). Very Low-Calorie Ketogenic Diet: A Safe and Effective Tool for Weight Loss in Patients with Obesity and Mild Kidney Failure. Nutrients.

[136] Moro T., Tinsley G., Longo G., Grigoletto D., Bianco A., Ferraris C., Guglielmetti M., Veneto A., Tagliabue A., Marcolin G. (2020). Time-restricted eating effects on performance, immune function, and body composition in elite cyclists: A randomized controlled trial. J. Int. Soc. Sports Nutr..

[137] Kitamura A., Seino S., Abe T., Nofuji Y., Yokoyama Y., Amano H., Nishi M., Taniguchi Y., Narita M., Fujiwara Y. (2021). Sarcopenia: Prevalence, associated factors, and the risk of mortality and disability in Japanese older adults. J. Cachexia Sarcopenia Muscle.

[138] Kim N.H., Park Y., Kim N.H., Kim S.G. (2021). Weight-adjusted waist index reflects fat and muscle mass in the opposite direction in older adults. Age Ageing.

[139] Beeri M.S., Leugrans S.E., Delbono O., Bennett D.A., Buchman A.S. (2021). Sarcopenia is associated with incident Alzheimer’s dementia, mild cognitive impairment, and cognitive decline. J. Am. Geriatr. Soc..

[140] Yee X.S., Ng Y.S., Allen J.C., Latib A., Tay E.L., Abu Bakar H.M., Ho C.Y.J., Koh W.C.C., Kwek H.H.T., Tay L. (2021). Performance on sit-to-stand tests in relation to measures of functional fitness and sarcopenia diagnosis in community-dwelling older adults. Eur. Rev. Aging Phys. Act..

[141] Konishi M., Kagiyama N., Kamiya K., Saito H., Saito K., Ogasahara Y., Maekawa E., Misumi T., Kitai T., Iwata K. (2021). Impact of sarcopenia on prognosis in patients with heart failure with reduced and preserved ejection fraction. Eur. J. Prev. Cardiol..

[142] Cheng K.Y.-K., Chow S.K.-H., Hung V.W.-Y., Wong C.H.-W., Wong R.M.-Y., Tsang C.S.-L., Kwok T., Cheung W.-H. (2021). Diagnosis of sarcopenia by evaluating skeletal muscle mass by adjusted bioimpedance analysis validated with dual-energy X-ray absorptiometry. J. Cachexia Sarcopenia Muscle.

[143] Merchant R.A., Seetharaman S., Au L., Wong M.W.K., Wong B.L.L., Tan L.F., Chen M.Z., Ng S.E., Soong J.T.Y., Hui R.J.Y. (2021). Relationship of Fat Mass Index and Fat Free Mass Index With Body Mass Index and Association With Function, Cognition and Sarcopenia in Pre-Frail Older Adults. Front. Endocrinol..

[144] Davis J.N., Pérez A., Asigbee F.M., Landry M.J., Vandyousefi S., Ghaddar R., Hoover A., Jeans M., Nikah K., Fischer B. (2021). School-based gardening, cooking and nutrition intervention increased vegetable intake but did not reduce BMI: Texas Sprouts—A cluster randomized controlled trial. Int. J. Behav. Nutr. Phys. Act..

[145] Lee Y.S., Hong N., Witanto J.N., Choi Y.R., Park J., Decazes P., Eude F., Kim C.O., Kim H.C., Goo J.M. (2021). Deep neural network for automatic volumetric segmentation of whole-body CT images for body composition assessment. Clin. Nutr..

[146] Akamatsu Y., Kusakabe T., Arai H., Yamamoto Y., Nakao K., Ikeue K., Ishihara Y., Tagami T., Yasoda A., Ishii K. (2022). Phase angle from bioelectrical impedance analysis is a useful indicator of muscle quality. J. Cachexia Sarcopenia Muscle.

[147] Petermann-Rocha F., Gray S.R., Forrest E., Welsh P., Sattar N., Celis-Morales C., Ho F.K., Pell J.P. (2022). Associations of muscle mass and grip strength with severe NAFLD: A prospective study of 333,295 UK Biobank participants. J. Hepatol..

[148] Wang Y., Zhang Y., Lane N.E., Wu J., Yang T., Li J., He H., Wei J., Zeng C., Lei G. (2022). Population-based metagenomics analysis reveals altered gut microbiome in sarcopenia: Data from the Xiangya Sarcopenia Study. J. Cachexia Sarcopenia Muscle.

[149] Tang T., Xie L., Hu S., Tan L., Lei X., Luo X., Yang L., Yang M. (2022). Serum creatinine and cystatin C-based diagnostic indices for sarcopenia in advanced non-small cell lung cancer. J. Cachexia Sarcopenia Muscle.

[150] Otsuka Y., Yamada Y., Maeda A., Izumo T., Rogi T., Shibata H., Fukuda M., Arimitsu T., Miyamoto N., Hashimoto T. (2022). Effects of resistance training intensity on muscle quantity/quality in middle-aged and older people: A randomized controlled trial. J. Cachexia Sarcopenia Muscle.

[151] Asvold B.O., Langhammer A., Rehn T.A., Kjelvik G., Grontvedt T.V., Sorgjerd E.P., Fenstad J.S., Heggland J., Holmen O., Stuifbergen M.C. (2023). Cohort Profile Update: The HUNT Study, Norway. Int. J. Epidemiol..

[152] Jeong S.-M., Lee D.H., Rezende L.F.M., Giovannucci E.L. (2023). Different correlation of body mass index with body fatness and obesity-related biomarker according to age, sex and race-ethnicity. Sci. Rep..

[153] Travers J., Romero-Ortuno R., Langan J., MacNamara F., McCormack D., McDermott C., McEntire J., McKiernan J., Lacey S., Doran P. (2023). Building resilience and reversing frailty: A randomised controlled trial of a primary care intervention for older adults. Age Ageing.

[154] Liu C., Cheng K.Y.-K., Tong X., Cheung W.-H., Chow S.K.-H., Law S.W., Wong R.M.Y. (2023). The role of obesity in sarcopenia and the optimal body composition to prevent against sarcopenia and obesity. Front. Endocrinol..

[155] Kiss N., Prado C.M., Daly R.M., Denehy L., Edbrooke L., Baguley B.J., Fraser S.F., Khosravi A., Abbott G. (2023). Low muscle mass, malnutrition, sarcopenia, and associations with survival in adults with cancer in the UK Biobank cohort. J. Cachexia Sarcopenia Muscle.

[156] Lin X., Song D., Shao T., Xue T., Hu W., Jiang W., Zou X., Liu N. (2024). A Multifunctional Biosensor via MXene Assisted by Conductive Metal-Organic Framework for Healthcare Monitoring. Adv. Funct. Mater..

[157] Feng Q., Besevic J., Conroy M., Omiyale W., Woodward M., Lacey B., Allen N. (2024). Waist-to-height ratio and body fat percentage as risk factors for ischemic cardiovascular disease: A prospective cohort study from UK Biobank. Am. J. Clin. Nutr..

[158] Reddy Y.N.V., Frantz R.P., Hemnes A.R., Hassoun P.M., Horn E., Leopold J.A., Rischard F., Rosenzweig E.B., Hill N.S., Erzurum S.C. (2025). Disentangling the Impact of Adiposity From Insulin Resistance in Heart Failure With Preserved Ejection Fraction. J. Am. Coll. Cardiol..

